# Decreased water temperature enhance *Piscine orthoreovirus* genotype 3 replication and severe heart pathology in experimentally infected rainbow trout

**DOI:** 10.3389/fvets.2023.1112466

**Published:** 2023-02-10

**Authors:** Juliane Sørensen, Argelia Cuenca, Anne Berit Olsen, Kerstin Skovgaard, Tine Moesgaard Iburg, Niels Jørgen Olesen, Niccolò Vendramin

**Affiliations:** ^1^Section for Fish and Shellfish Diseases, National Institute for Aquatic Resources, Technical University of Denmark, Kgs. Lyngby, Denmark; ^2^Section of Aquatic Biosecurity Research, Norwegian Veterinary Institute, Bergen, Norway; ^3^Department of Biotechnology and Biomedicine, Technical University of Denmark, Kgs. Lyngby, Denmark

**Keywords:** *Piscine orthoreovirus* genotype 3 (PRV-3), double stranded RNA (dsRNA) virus, temperature, immune response, rainbow trout, RAS

## Abstract

*Piscine orthoreovirus* genotype 3 (PRV-3) was first discovered in Denmark in 2017 in relation to disease outbreaks in rainbow trout (*Oncorhynchus mykiss*). While the virus appears to be widespread in farmed rainbow trout, disease outbreaks associated with detection of PRV-3 have only occurred in recirculating aquaculture systems, and has predominantly been observed during the winter months. To explore the possible effects of water temperature on PRV-3 infection in rainbow trout, an *in vivo* cohabitation trial was conducted at 5, 12, and 18°C. For each water temperature, a control tank containing mock-injected shedder fish and a tank with PRV-3 exposed fish were included. Samples were collected from all experimental groups every 2nd week post challenge (WPC) up until trial termination at 12 WPC. PRV-3 RNA load measured in heart tissue of cohabitants peaked at 6 WPC for animals maintained at 12 and 18°C, while it reached its peak at 12 WPC in fish maintained at 5°C. In addition to the time shift, significantly more virus was detected at the peak in fish maintained at 5°C compared to 12 and 18°C. In shedders, fish at 12 and 18°C cleared the infection considerably faster than the fish at 5°C: while shedders at 18 and 12°C had cleared most of the virus at 4 and 6 WPC, respectively, high virus load persisted in the shedders at 5°C until 12 WPC. Furthermore, a significant reduction in the hematocrit levels was observed in the cohabitants at 12°C in correlation with the peak in viremia at 6 WPC; no changes in hematocrit was observed at 18°C, while a non-significant reduction (due to large individual variation) trend was observed at cohabitants held at 5°C. Importantly, *isg15* expression was positively correlated with PRV-3 virus load in all PRV-3 exposed groups. Immune gene expression analysis showed a distinct gene profile in PRV-3 exposed fish maintained at 5°C compared to 12 and 18°C. The immune markers mostly differentially expressed in the group at 5°C were important antiviral genes including *rigi, ifit5* and *rsad2* (viperin). In conclusion, these data show that low water temperature allow for significantly higher PRV-3 replication in rainbow trout, and a tendency for more severe heart pathology development in PRV-3 injected fish. Increased viral replication was mirrored by increased expression of important antiviral genes. Despite no mortality being observed in the experimental trial, the data comply with field observations of clinical disease outbreaks during winter and cold months.

## 1. Introduction

*Piscine orthoreovirus* is a double stranded RNA (dsRNA) virus, with a genome consisting of 10 segments (1–3). The virus is non-enveloped, with a double protein capsid in icosahedral structure (1).

Three genotypes of PRV have been described (4, 5), each with different host tropism:

PRV-1, which infects Atlantic salmon (*Salmo salar*), has been shown to be the causative agent of heart and skeletal muscle inflammation (HSMI) in Norway (6). Importantly, different strains representing different geographic regions or time of detection show different capabilities of inducing HSMI (6, 7). PRV-1 has also been linked to alteration in color of Atlantic salmon fillet (8).

PRV-2, which has been shown as the putative causative agent of erythrocytic inclusion body syndrome (EIBS) in coho salmon (*Oncorhynchus kisutch*) in Japan (9).

PRV-3, which primarily infects rainbow trout (*Oncorhynchus mykiss*), was first discovered in Norway in 2013 in a disease outbreak in farmed rainbow trout (10). It was then detected in Denmark in farmed rainbow trout in late 2017 and early 2018 in field cases with increased mortality and signs of abnormal swimming behavior (3, 11). As a result, a surveillance program was conducted in Denmark including 53 farms, both flow-through and recirculated aquaculture systems (RAS), in order to assess the spread of PRV-3 within Danish aquaculture (4). The study showed that ~72% of the farms in the surveillance program were positive for PRV-3 at least once for the duration of the program (~1.5 years). Notably, PRV-3 genotypes divide into two clades, which are geographically distinct. PRV-3a has so far only been detected in Norway, while PRV-3b has been detected in exchange and aeration, has shown no significant Europe and Chile (4). However, only RAS farms experienced cases of increased mortality in association with PRV-3 detection in Denmark (4). The occurrence of PRV-3 infection is often associated with other production pathogens [e.g., *Flavobacterium psychrophilum, Renibacterium salmoninarum*, and infectious pancreatic necrosis virus (IPNV); personal communication]. In the surveillance conducted in 2017–2019, while PRV-3 was detected at RAS farms throughout the year, all 14 disease outbreaks were recorded in December to April where the water temperature was low (4).

Experimental infection both with infected blood and purified viral particles have depicted the pathogenesis of the virus at the temperature of 12°C, and showed its capability of inducing heart pathology. However, private practitioners and farmers report more severe disease outbreaks where PRV-3 is detected during winter and spring, when the water temperature is lower (12, 13).

Experimental infection trials with PRV-1, -2, and -3 have yet to replicate the mortality observed in field cases despite observations of heart inflammation and anemia (6, 9, 13). In some instances, reduced survival is not observed experimentally even when fish are exposed to additional stressors beyond PRV challenge (6, 9, 14–20), pointing toward a complex host-pathogen-environment interaction to achieve clinical disease development.

Fish are poikilothermic organisms, with temperature having a measurable effect on energy metabolism (21). Notably, the host-pathogen interaction is also affected by temperature, either by the effect of temperature on the immune status of fish (22, 23), by affecting the capability of the pathogen to replicate at different environmental temperature, or a combination of both (24). Additionally, behavioral fever has been studied in fish; it induces fish to move to warmer areas as a response to infections (particularly viruses) (25). The increased water temperature has been shown to both improve immune response and modulate replication of the pathogen (26). This behavior has been reported for fish affected by HSMI (27, 28), and in a study which has reported epigenetic changes of the immune response in Atlantic salmon exposed to IPNV (29). Considering that disease outbreaks in Danish farms associated with PRV-3 have mostly been reported during winter, we attempt to dissect the host-pathogen-environment interaction by examining the effect of water temperature on PRV-3 infection kinetics.

By exposing rainbow trout to PRV-3 in a cohabitation trial at three different temperatures (5, 12, and 18°C), we have elucidated its effect on viral load kinetics, hematocrit, heart histopathology, and immune gene expression. The results of the experiment align with field observations, supporting the fact that low water temperature enhance PRV-3 replication and severe heart pathology.

## 2. Materials and methods

### 2.1. Production and preparation of inoculum

The experiment was conducted under license number 2019-15-0201-00159, and the experimental protocols were approved by the Danish Animal Research Authority. The PRV-3b isolate (DK/PRV315, accession number MW012855.1) used for inoculum was propagated *in vivo* following the procedure as previously described (3).

Briefly, Specific Pathogen Free (SPF) rainbow trout (average size of 30 g) were anesthetized in water containing benzocaine (80 mg/L, Sigma) and injected i.p. with 0.1 mL homogenized blood cell pellet from PRV-3b infected fish diluted 1:3 (v/v) in L-15 medium. The PRV-3 levels were monitored weekly by non-lethal blood sampling from five fish, which were marked by clipping of the adipose fin to avoid repeated sampling of the same fish. At 3 weeks post challenge (WPC), all of the fish were euthanized by immersing fish in water containing high concentration of benzocaine (800 mg/L). Blood was collected in heparin tubes, tested for PRV-3 levels by RT-qPCR (see below), and stored at 4°C.

The PRV-3 positive inoculum for the experiment consisted of 10 mL PRV-3b positive blood diluted in 10 mL L-15 medium and 500 μL gentamycin (Life technologies, Carlsbad, CA, USA). This inoculum was tested by RT-qPCR, with a Ct value of 29.4. The mock inoculum consisted of 5 mL blood from naive fish diluted in 15 mL L-15 medium and 500 μL gentamycin. Mock inoculum was confirmed negative by RT-qPCR.

### 2.2. Experimental Trial

The experiment was conducted under license as previously described.

Rainbow trout were obtained from eyed eggs provided by a Danish commercial fish farm registered officially free of infectious pancreatic necrosis virus (IPNV), infectious hematopoietic necrosis virus (IHNV), viral hemorrhagic septicemia virus (VHSV), and *Renibacterium salmoninarum* (bacterial kidney disease, BKD). After disinfection procedures using iodine, the eggs were hatched and grown in the wet laboratory facilities of section for fish and shellfish diseases, DTU Aqua, Kgs. Lyngby, Denmark) in recirculating and UV disinfected tap water (12°C).

**Figure 1:**
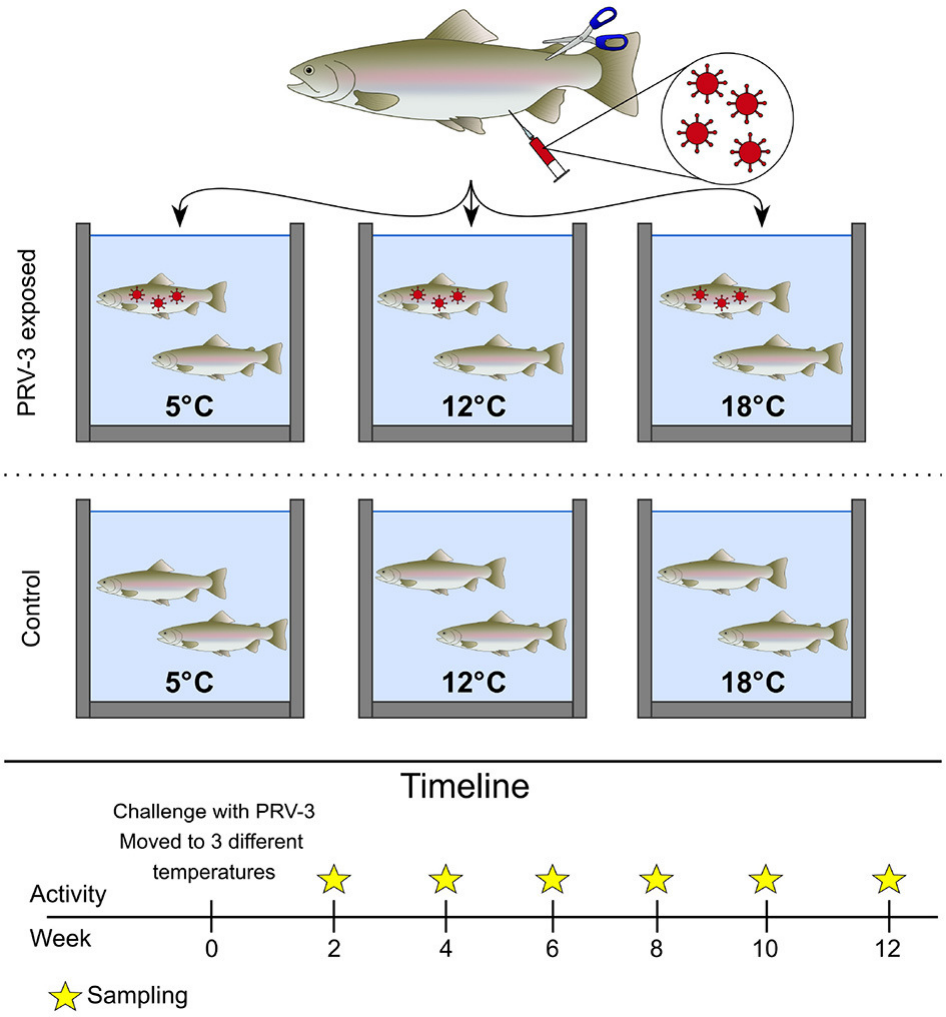
Experimental trial setup. The trial was conducted as a cohabitation trial, with one control and one PRV-3 exposed tank per temperature. The fish were kept at 12°C until i.p., injection, and were then moved to the respective temperatures (5, 12, and 18°C). Samples were collected every 2nd week post challenge according to the sampling table.

Before beginning the trial, SPF rainbow trout were moved into the high containment infection facility at EURL. Six-hundred fish of ~15 g were divided into six 180 L tanks run with 15 L/h flow-through fresh water renewal at the following conditions: L:D 12:12, stocking density below 60 kg/m3, and feeding at 1% of the biomass. The water temperature was set to 5, 12, and 18°C, with two tanks for each temperature (see Figure 1). The temperature was monitored throughout the trial (±2° in all temperatures). The experimental setting, including biomass, feeding ration, water exchange and aeration, has shown no significant difference in the level of oxygen and saturation in the water at the three temperatures. Fish were not acclimatized before the start of the experiment, but all started at 12°C before treatment.

The experimental trial was conducted as a cohabitation trial with a 50:50 distribution of shedders (intraperitoneal injection, i.p.) and cohabitants. Shedders were anesthetized by immersion in benzocaine-containing water (80 mg/L), and i.p. injected with 40 μL PRV-3b or mock inoculum. To later identify shedders, the adipose fin was clipped.

To confirm that the fish were infected with PRV-3b, RNA was extracted from the fish (heart tissue) at the peak of viral load at each temperature, with magnetic bead-based extraction using Indimag Pathogen Kit in an IndiMag48 automated extraction system (Indical Biosciences) according to manufacturer's recommendations.

RT-PCR targeting the S1 segment (10) of PRV-3 was performed using OneStep RT-PCR kit (Qiagen) according to manufacturer's recommendations. The PCR products were separated by electrophoresis using 1.2% E-gels (ThermoFisher Scientific) and E-gel powerbase version 4 (Invitrogen) as instructed by the manufacturer. PCR fragments were cleaned with the QIAquick PCR Purification kit (Qiagen) according to manufacturer's recommendations, and Sanger sequencing was performed by Eurofins Genomics (Ebersberg, Germany). Sequence analysis was done using CLC Main Workbench 8 (version 11.0.1) and subsequently run through nucleotide BLAST, confirming that the experimental fish were infected with PRV-3b.

### 2.3. Sample collection

Samples were collected every 2nd week post challenge from weeks 2 to 12. At each sampling point, 6:6 and 3:3 shedders:cohabitants from PRV-3b exposed and control groups, respectively, were euthanized in benzocaine-containing water (800 mg/L) and then sampled. Blood was collected from the caudal vein in heparin-coated 1.5 mL tubes (Eppendorf) from the cohabitants. From all experimental groups, heart, spleen, and kidney were collected in 1.5 mL tubes containing 500 μL RNAlater (Invitrogen). Additionally, heart, spleen, gill, brain, kidney, and intestine were collected in 10% neutral buffered formalin (4% formaldehyde (VWR International A/S) for histopathological examination. For further details, see sampling plan in Table 1.

**Table 1:** Sampling plan for all experimental groups (5, 12, and 18°C).

**Group**	**Number of fish**	**Fish sampled per time point**	**Sampling points (WPC)**	**Sample**	**Purpose**
Negative control	50 shedders (mock injected)	3	2, 4, 6, 8, 10, 12	Heart	PRV-3 qPCR Viral load
	50 cohabitants	3		Spleen	Immune gene expression
PRV-3	50 shedders (PRV-3 exposed)	6		Kidney	Backup
				Blood	hematocrit
	50 cohabitants	6		Organs	Histopathology

### 2.4. Improved RT-qPCR assay for PRV-3 detection

Based on previous sequence analysis of isolates from Denmark, a mismatch was observed in the region of S1 segment targeted by the RT-qPCR developed by Olsen et al. (10). In order to ensure optimal detection, a new assay was designed targeting the L1 segment. See Table 2 for primer and probe sequences. The new assay was tested against a panel of fish pathogens for specificity, and against both PRV-3 subgenotypes (PRV-3a and -3b) and an artificial control (PRV-3 gBlock, IDT, see Supplementary Table S1) to ensure equal and efficient detection of both.

**Table 2:** Sequences and final concentration of primers and probes for PRV-3 detection by RT-qPCR.

**Primer/probe**	**Sequence**	**Concentration (nM)**	**References**
L1PRV3-951F	TACAGGTCGTGTTCCCGTTG	700	This paper
L1PRV3-1042R	TCCAGCCACGAGGTAGATCA	700	This paper
L1PRV3-1003 probe	/56-FAM/TTCAGGTTG/ZEN/ GATGGAGCGCG/3IABkFQ/	200	This paper
ELF1a_FW	CCC CTC CAG GAT GTC TAC AAA	200	(30)
ELF1a_BW	CAC ACG GCC CAC GGG TAC T	200	(30)
ELF1a_P	/5HEX/-ATC GGC GGT/ZEN/ATT GGA AC-/3IABkFQ/	50	(31)

### 2.5. Virus detection by RT-qPCR

Approximately 25 mg of heart tissue was homogenized in 600 μL PBS with a 5 mm stainless steel bead (Qiagen) for 2 min at 25 Hz on TissueLyzer II (Qiagen). The samples were centrifuged at 14,000 x g for 5 min at 4°C, and total RNA was extracted from 200 μL supernatant using the IndiMag Pathogen extraction (Indical Biosciences) kit previously described.

Viral load was assessed by RT-qPCR using TaqPath 1-Step Master Mix (Applied Biosystems) according to manufacturer's recommendations, using 5 μL RNA template in a total volume of 25 μL. As the starting amount of RNA template was not standardized among samples, the L1PRV3 assay was multiplexed with ELF1a assay (30, 31) in the concentrations shown in Table 2, with the following thermal profile: 30 min at 50°C, 15 min at 95°C, 50 cycles of 15 s at 94°C and 1 min at 60°C.

Data was collected and analyzed using mxPro–mx3005P v4.10 Build 389, Schema 85 (Stratagene). The threshold was set using an inter-plate callibrator (PRV-3 positive tissue PCR control) for both PRV-3 and ELF1A. Data was exported to Excel 2016 (Microsoft), and Ct values of PRV-3 were normalized:


Ct(PRV-3)-Ct(ELF1a)=dCt

Statistical analyses for virus RNA load was performed on log2 transformed data of PRV-3 positive samples at selected time points.

### 2.6. Immune gene expression by high throughput microfluidic qPCR

Samples for immune gene expression analyses were selected based on the virus load in each experimental group, selecting samples collected before, during, and after highest virus RNA load. For shedders, samples collected at all time points at 5°C were selected, and at 12 and 18°C samples collected at 2–6 WPC were selected. For cohabitants, 8–12 WPC was selected for 5°C, and 2–8 WPC was selected for 12 and 18°C.

For immune gene expression analyses, RNA was extracted from spleen with RNeasy Mini Kit (Qiagen) using QiaCube (Qiagen) according to manufacturer's recommendations. Briefly, ~25 mg tissue was homogenized in 700 μL RLT buffer with a 5 mm stainless steel bead (Qiagen) for 2 min at 25 Hz on TissueLyzer II (Qiagen). The lysate was centrifuged for 5 min at 4°C at 14,000 x g, and 600 μL supernatant was transferred to a clean tube for automatic extraction on QiaCube.

The concentration was measured by Nanodrop (Nanodrop 1000, Thermo Scientific), and the quality of the RNA was checked on a Bioanalyzer 2100 (Agilent) using RNA 6000 Nano Kit (Agilent) according to manufacturer's recommendations with an input of ~100 ng total RNA. RNA integrity number (RIN) above 6 was accepted for downstream applications.

cDNA synthesis was performed using QuantiTect Reverse Transcription Kit (Qiagen) according to manufacturer's recommendations and as previously described (32), with an input of 500 ng total RNA. As control for genomic DNA (gDNA) contamination, samples with high RIN value were chosen as non-reverse transcription controls. After cDNA synthesis, all samples were diluted 2 μL cDNA in 18 μL Low EDTA TE-buffer (VWR).

Pre-amplification was performed using TaqMan PreAmp Master Mix (Applied Biosystems) as previously described (32). Briefly, pre-amplification was performed with a 20 μM primer mix containing all assays as shown in Table 3, with the following thermal cycling conditions: 95°C for 10 min, 20 cycles: 95°C for 15 s and 60°C for 4 min. After pre-amplification, all samples were treated with Exonuclease 1 (4 units/μL, New England Biolabs).

**Table 3:** Primers used for immune gene expression in spleen tissue.

**Target**	**Primer name**	**Sequence**
	RBT_mx_OI_73F	CTCATCTCAGCACACTTTATGATG
mx	RBT_mx_OI_73R	GGCAGGGATTTCTCGATATG
	RBT_cd4_OI_89_F	CACACTGAAGATCGAGCGAGT
cd4	RBT_cd4_OI_89_R	GGATGAGGAGGAGGACGAAT
	RBT_cd8A_77_F	CCACGACGACTACACCAATG
cd8	RBT_cd8A_77_R	CTTTCCCACTTTGCACGACT
	RBT_ifng_70_F	ACACCGGGAAGTTGATCTTG
ifng	RBT_ifng_70_R	CTCCCCCAATCCTAACCTTC
	RBT_rsad2_OI_78_F	GCTGGAAGGTGTTCCAGTGT
rsad2	RBT_rsad2_OI_78_R	GGTCGCTGATGAGAAACCTC
	RBT_ifnc3_OI_78_F	CACAGTTGAGCAGCAGTGGT
ifnc3	RBT_ifnc3_OI_78_R	GGTCGTCAGCTCCAAACAAT
	RBT_tnf_OI_88_F	CTGGCAACGATGCAGGA
tnf	RBT_tnf_OI_88_R	CGGCAATCTGCTTCAATGTA
	RBT_il1b_OI_82_F	CAGCAGCTACCACAAAGTGC
il1b	RBT_il1b_OI_82_R	GGCTACAGGTCTGGCTTCAG
	RBT_irf8_OI_90_F	CGACGCCTCTATCTTCAAGG
irf8	RBT_irf8_OI_ 90_R	AGCCTGGTCTTCCATGTAGC
	RBT_MHCclassII_ 100_F	CAGGTTTCTACCCCAGTGGA
mhc class II	RBT_MHCclassII_ 100_R	CCATCATCGTTTGGGAGAGT
	RBT_gzma_82_F	CAAGACCAGGGTGGACTCAT
gzma	RBT_gzma_82_R	GAACGACACGACTCCCCTTA
	RBT_DDX58_105_F	ACTGAGATGCTCCGCAAGAT
rigi	RBT_DDX58_105_R	CTGGTAGCTGCCTTCAGTCC
	RBT_isg15_85_F	ATCCTGAATGCAGGCCATAG
isg15	RBT_isg15_85_R	CAGAGGCTGTCAGGTGTCAA
	RBT_tlr3_83_F	CTCTAACGGCAACCAGAAGC
tlr3	RBT_tlr3_83_R	CTCTCCCCAGCATCAGAGTC
	RBT_cxcl10/IP-10/11-1_107_F	ATCCATGACCAACACGATGA
cxcl10	RBT_cxcl10/IP-10/11-1_107_R	ACAGGCACCGAGCTTTAGAA
	RBT_saa_i_78_F	CCCTCGTTGTAGGAGCTCAA
saa	RBT_saa_i_78_R	CACGCCACATGTCTTTAGCA
	RBT_hp_84_F	GCCTTGATCTTGTAAAACTCTCTCAA
hp	RBT_hp_84_R	TCAACATCGGAAGACATACTCAATC
	RBT_ifit5_73_F	AGAGAGGTGGCAGGCTAACA
ifit5	RBT_ifit5_73_R	CCTCTCCTGTTTGAGGAACG
	RBT_ifi44_OI_76_F	GGAAAGCTGAGAGGAGAAAGG
ifi44	RBT_ifi44_OI_76_R	GCCTGACCCACAGAACTGAT
	RBT_irf1_OI_96_F	CACCCCACAGACTATGAAGACAG
irf1	RBT_irf1_OI_96_R	GCTCAGGAACCTCTTGTCGT
	RBT_csf1r_OI_71_F	GCAGCCAAGAACTGTATTCACC
csf1	RBT_csf1r_OI_71_R	TTAGCCACATGGAGGTCTGTC
	RBT_actb_OI_84_F	GAAGATGACCCAGATTATGTTTGAG
actb	RBT_actb_OI_84_R	GAGGCGTACAGGGACAACAC
	RBT_hprt1_OI_73_F	CCTTGACAGGACAGAGAGGCTA
hprt1	RBT_hprt1_OI_73_R	GCAGAGGGCCACGATATG
	RBT_eef1a1_OI_76_F	CGGTGGTATTGGAACTGTACCT
eef1a1	RBT_eef1a1_OI_76_R	GGCGAAGGTGACGATCATA

“OI” indicates that the forward primer has been designed to cross an intron.

High throughput microfluidic qPCR on the pre-amplified, exonuclease treated cDNA samples was performed using 192.24 Dynamic Array IFC (Fluidigm/Standard Biotools) as previously described (33).

Briefly, 21 genes of interest (GOI) were selected along with the references genes listed in Table 3. The 192.24 Dynamic Array combines 192 samples with 24 different assays, resulting in 4,608 individual qPCR reactions running simultaneously.

Raw data analysis was performed in the Fluidigm Real-Time PCR Analysis software (version 4.7.1, build 20200930.1707, Fluidigm Corporation), and then exported to GenEx (version 7, MultiD Analyses AB).

To calibrate for variation between IFCs (integrated fluidic circuits), four samples were selected as interplate calibrators and included in each IFC run. After interplate calibration, all data was efficiency corrected based on the efficiency for each individual assay. To identify the most suitable reference genes, GeNorm and GeNormFinder in GenEx were used. Of the three reference genes, *hprt1* and *eef1a1* were selected and the geometric mean of the two selected reference genes was used to normalize all genes.

### 2.7. Hematocrit

Hematocrit (hct) was measured by centrifuging an aliquot of heparinized full blood in glass microhematocrit tubes (manufacturer) at 2,000 x g for 10 min. The hematocrit value (% red blood cells) was read on a visual analogue scale. The remaining blood was centrifuged at the same speed and time for separation of plasma and blood cells.

### 2.8. Histopathology of heart

According to Olsen et al. (10),the heart was the organ most consistently affected in diseased rainbow trout naturally infected with PRV-3. Accordingly, heart was chosen as the indicator of tissue effect visible by light microscopy. Heart sampled from all experimental groups (see Table 1) were fixed in 10% neutral buffered formalin, and processed as previously described (10). The slides were assessed blinded. Histopathological findings were scored as none or very sparse (0–0.5), mild (1), moderate (1.5), and severe (2) (see Supplementary Figure S5).

### 2.9. Statistical analysis

Statistical analyses and plots of virus RNA load, hematocrit, and histopathology was performed in Graphpad Prism 9 [version 9.4.1 (681)]. Kruskuall-Wallis and Mann-Whitney test was selected for comparison between groups where relevant. Area under the curve (AUC) was used to assess the differences in virus load in shedders at two to eight WPC, and cohabitants at four to ten WPC at 18 and 12°C.

Data analysis of immune gene expression data was performed as previously described (34). Briefly, for fold-change calculation of immune genes between control and PRV-3 exposed groups, relative quantities (RQ) of the PRV-3 exposed groups were scaled according the control of the corresponding control group, setting the mean value of the controls to 1. Statistical analyses were performed in Excel 2016 on log2 transformed non-scaled RQ values. T-test was selected, as gene expression values are assumed to be normally distributed within the given groups. Only genes with both *p* < 0.05 in addition to fold change>2.0 or < 0.5 were considered significantly up- or down-regulated. Heatmaps were created in R (version 4.0.5) with Rstudio (version 1.4.1106), using the pheatmap package, and were based on the median value of the log2 transformed RQ values for each group. Graphs of individual genes were made based on non-scaled RQ values in Graphpad Prism 9.

Pearson's correlation was calculated using GenEx (version 7, MultiD Analyses AB).

## 3. Results

### 3.1. New RT-qPCR assay performance

To assess the specificity and sensitivity of the newly developed PRV-3 qPCR assay targeting the L1 segment, the assay was tested against a series of pathogens, and standard curves based on both subgenotypes of PRV-3 along with the PRV-3 gblock (artificial control). Of the following pathogens, none besides PRV-3 were detected by the assay: *R. salmoninarum*, CEV, *A. invadans*, IHNV, IPNV, ISAV, KHV, MLO/RMS, NODA virus, OMV, PFRV, *P. salmonis, T. bryosalmonae*, PMCV, SGPV, PRV-1, PRV-2, SAV, SVCV, TSV, VHSV, and WSSV.

Limit of detection was 7.6 copies per reaction (tested in quadruplicates) at Ct 31.

### 3.2. Virus load across temperatures

**Figure 2:**
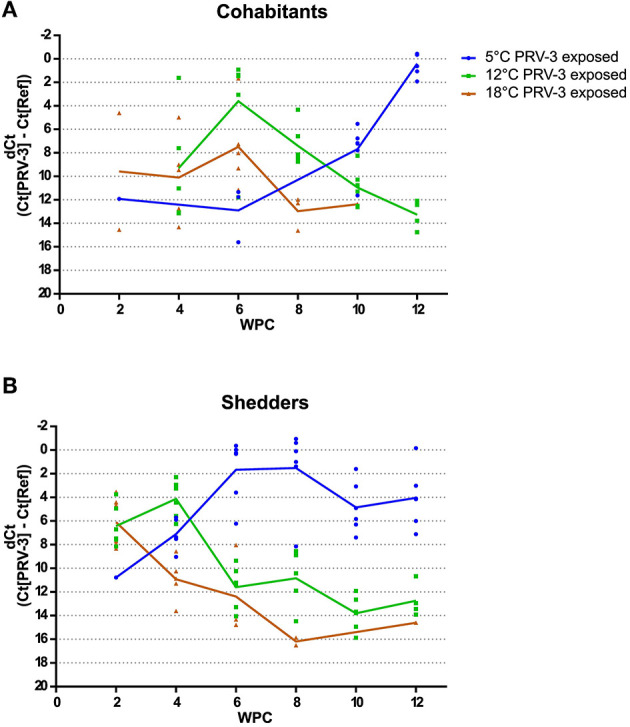
PRV-3 virus load shown as dCt [= Ct(PRV-3)-Ct(Reference gene)] for **(A)** cohabitants, and **(B)** shedders. Only individuals that tested positive are shown on the graphs. In cohabitants, a significant difference in virus load was detected at 5°C at 12 WPC compared to 6 WPC at 12 and 18°C (time point of highest virus load, *p* = 0.024 and *p* = 0.009, respectively). Similar tendency was observed in the shedders: at 5°C 8 WPC, a significantly higher amount of virus was detected compared to 2 WPC at 18°C and 4 WPC at 12°C (*p* = 0.041 and *p* = 0.026, respectively).

Figure 2 shows the dCt values of PRV-3 positive fish for both cohabitants (Figure 2A) and shedders (Figure 2B) measured in heart tissue. Table 4 shows the number of PRV-3 positive fish in the PRV-3 exposed groups at the different temperatures and time points.

**Table 4:** Number of PRV-3 positive fish at a given time-point and temperature.

**WPC/ temperature**	**2**	**4**	**6**	**8**	**10**	**12**	**Total no. of positive fish**
**Cohabitants**							
5°C	1	0	3	0	6	6	16/36
12°C	0	5	6	6	6	4	27/36
18°C	2	5	5	3	1	0	16/36
**Shedders**							
5°C	1	5	6	6	6	6	30/36
12°C	6	6	6	5	6	4	33/36
18°C	6	5	3	2	0	1	17/36

In cohabitants (see Figure 2A), virus RNA load peaked at 6 weeks post challenge for both 12 and 18°C, with a lower amount at 18°C (not statistically different, *p* = 0.16 Mann-Whitney test). At this time point, six out of six and five out of six fish at 12 and 18°C, respectively, were positive for PRV-3. From 6 to 12 WPC, the virus RNA load steadily decreased in both groups; four and zero out of six fish were positive for PRV-3 at 12 WPC at 12 and 18°C, respectively (see Table 4).

At 5°C, only four cohabitant fish had tested positive up until 10 WPC, and at very low virus levels. However, at 10 weeks post challenge, all six fish were positive with medium-high virus load. At 12 WPC, six out of six fish were positive with high virus load. Comparing the time point with highest virus load at the three different temperature (6 WPC for 12 and 18°C, and 12 WPC for 5°C), there was significantly more virus detected at 5°C compared to 12 and 18°C (*p* = 0.024 and *p* = 0.009, respectively).

In shedders (see Figure 2B), fish kept at 18 and 12°C had high virus load with all six fish testing positive for PRV-3 at 2 and 4 WPC, respectively, after which the virus load decreases. At the end of the experiment, one and four fish at 18 and 12°C were PRV-3 positive at low virus levels, respectively.

Shedders at 5°C experienced a time delay in virus load. Here, high virus load is detected at 6 WPC where all six fish tested positive. Interestingly, for the remainder of the experiment, all shedders kept at 5°C were PRV-3 positive, and at high virus load. The fish seem unable to clear the infection, unlike what is typically observed at higher temperatures. Comparing the time points with highest virus load at the different conditions, fish kept at 5°C at 8 WPC had significantly higher virus load compared to fish at 18°C at 2 WPC (*p* = 0.041), and compared to 12°C at 4 WPC (*p* = 0.026). No significant difference was observed between shedders maintained at 12 and 18°C (*p* = 0.818).

### 3.3. Hematocrit fluctuations due to temperature and PRV-3 infection

**Figure 3:**
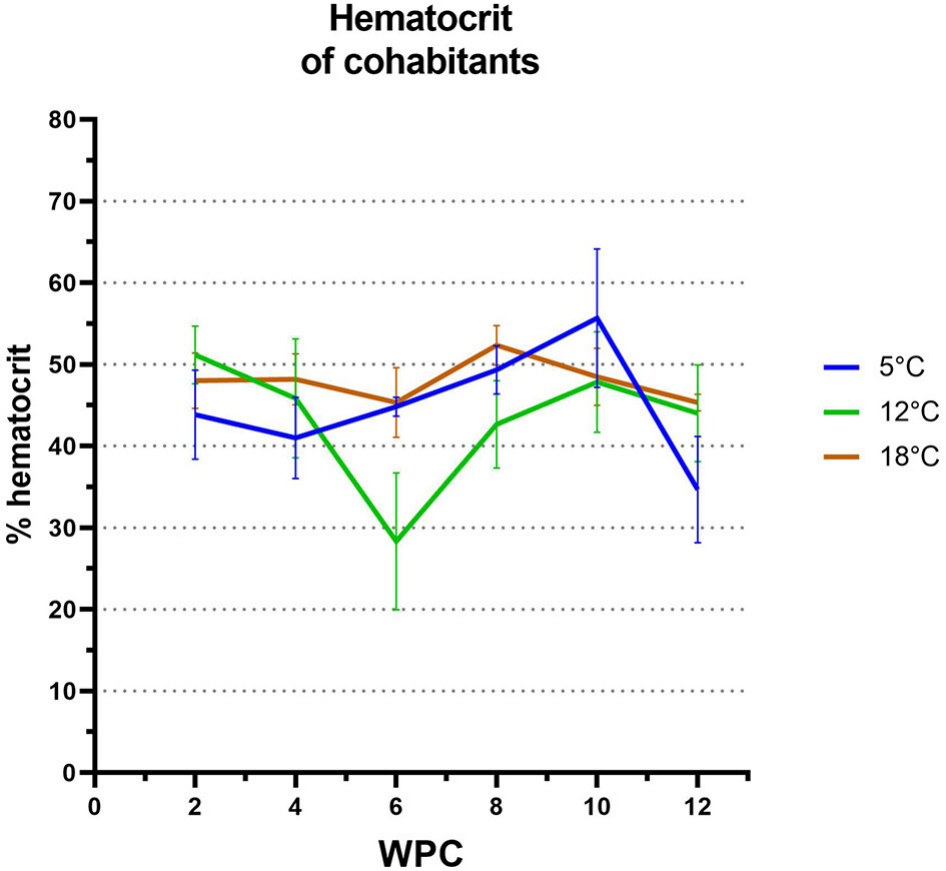
Hematocrit levels of cohabitants exposed to PRV-3. Significant difference observed in PRV-3 exposed cohabitants at 12°C compared to 5 and 18°C at six WPC (*p* = 0.05 and *p* = 0.026, respectively). Likewise, a significant difference was observed at 5°C compared to 12 and 18°C at 12 WPC (*p* = 0.026 and *p* = 0.0096, respectively). Error bars show SD.

Cohabitants exposed to PRV-3 kept at 12°C experienced a significant reduction in hct levels compared to cohabitants kept at 5 and 18°C at 6 weeks post challenge (*p* = 0,005 and *p* = 0.026, respectively), corresponding to the time of highest virus load in the 12°C group. Here, a reduction from ~50 to 30% hct was observed (see Figure 3).

Similar was observed for the cohabitants exposed to PRV-3 kept at 5°C: a significant reduction in hct occurred at 12 weeks post challenge, corresponding to the time point of highest virus load in this group (*p* = 0.0268 and *p* = 0.0096 for 12 and 18°C, respectively).

No reduction in hct was observed at 18°C in PRV-3 exposed cohabitants.

However, when comparing to non-infected control fish at each temperature, there was no significant difference from control to exposed at 5°C due to the large variation both within the control and the exposed group (data not shown). Additionally, the mock-infected fish at 18°C had a significantly lower hct at the time of highest virus load compared to the PRV-3 exposed fish at this temperature (data not shown).

### 3.4. Histopathology in the heart

**Figure 4:**
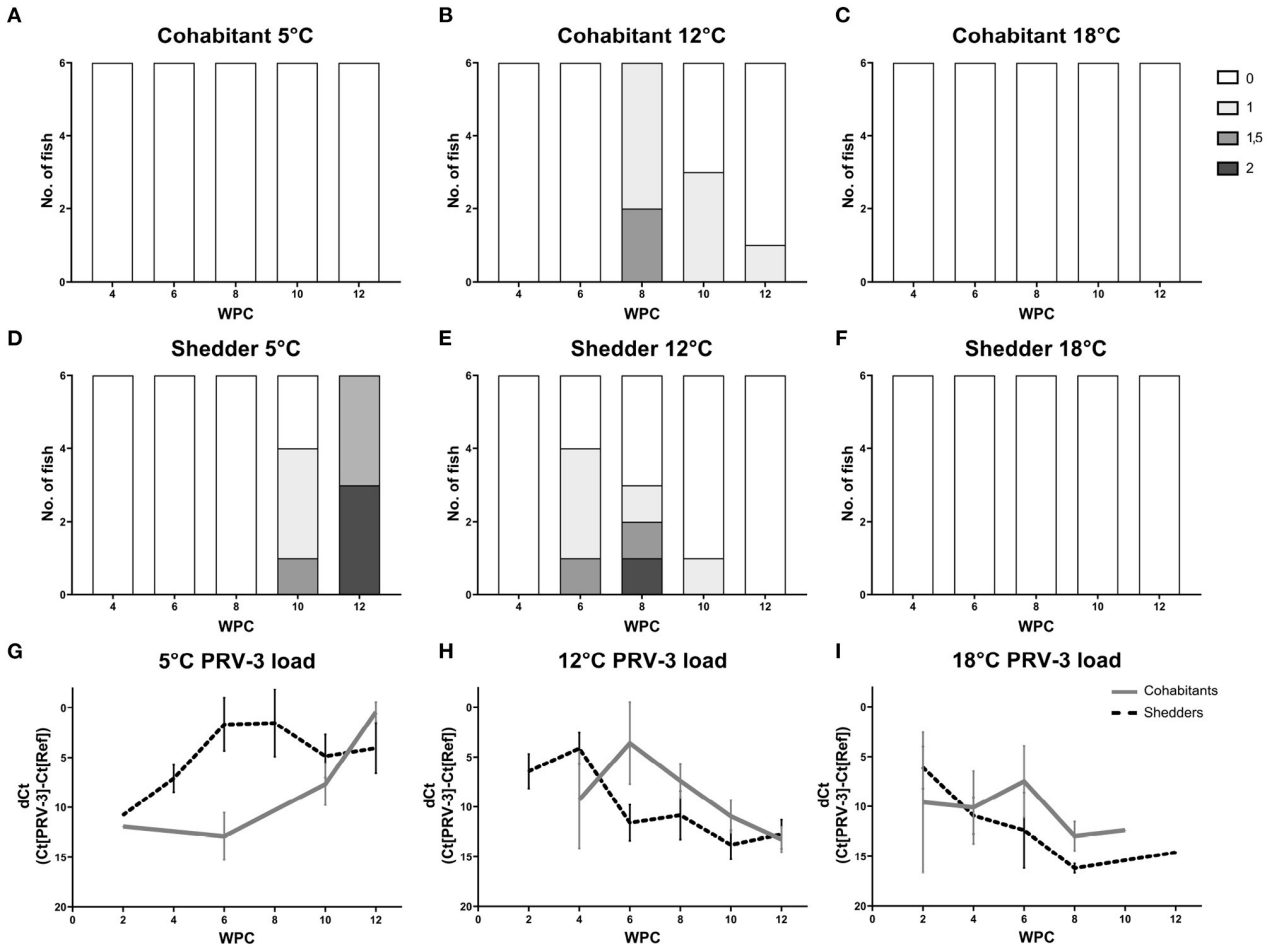
Heart histopathology of shedders **(A–C)** and cohabitants **(D–F)** of PRV-3 exposed fish in comparison to dCt values **(G–I)**. Histoscore 0 = no lesions, 1 = mild, 1.5 = moderate, 2 = severe. Error bars show SD.

Histopathological findings in hearts consistent with PRV-3 associated pathology described earlier (10, 12) were observed in the cohabitants at 12°C 2 weeks post peak load of virus. Six out of six fish had heart lesions at 8 WPC, with highest histoscore of 1.5 (see Figure 4B).

Cohabitants kept at 5°C did not show any signs of lesions in the heart (Figure 4A). However, heart lesions were seen in the shedders at 10 WPC, i.e., 4 weeks post peak virus load (five out of six fish, Figure 4D). At 12 WPC, all six 5°C shedders had heart lesions, and to severe degrees (3:3 with histoscores 1.5 and 2, Figure 4E). This is compared to shedders at 12°C at 8 WPC, at which three out of six fish had heart lesions, 1:1:1 with histoscores 1, 1.5, and 2. No statistically significant difference between heart lesions in the shedders at 12°C 8 WPC and 5°C 12 WPC was observed, however there was a trend toward more severe lesions at 5°C (*p* = 0.0563).

No heart histopathology was observed in cohabitants and shedders kept at 18°C during the experiment (see Figures 4C, F).

### 3.5. Immune gene profiles

In order to assess the impact of water temperature on the immune gene status of both control and PRV-3 exposed fish, 21 different immune genes covering both the innate and the adaptive immune system were selected for immune gene expression by microfluidic qPCR (see Table 3 for a full list of the genes and primer sequences). Typically, in our experimental facility, standard *in vivo* studies in rainbow trout are conducted at 12°C, and therefore this group was considered the baseline in this study.

#### 3.5.1. Temperature induced regulation of anti-viral genes in non-infected control fish

In mock-injected shedders, fish at all three temperatures had a relatively similar immune gene expression profile forming a single cluster (see Figure 6). Overall, the immune profile of PRV-3 exposed fish differed from the relatively homogeneous profile of control fish regardless the temperature. However, significant differences in the immune profile expression of certain genes were observed between 5 and 18°C controls: Up-regulation of *cd4, rigi, isg15, saa, ifit5* and *ifi44* was observed at 18°C compared to 5°C (*p* < 0.01). At 5°C, down-regulation of *ifnc3* was observed compared to 12°C (*p* = 0.001). No significant differences between 12 and 18°C were observed.

**Figure 5:**
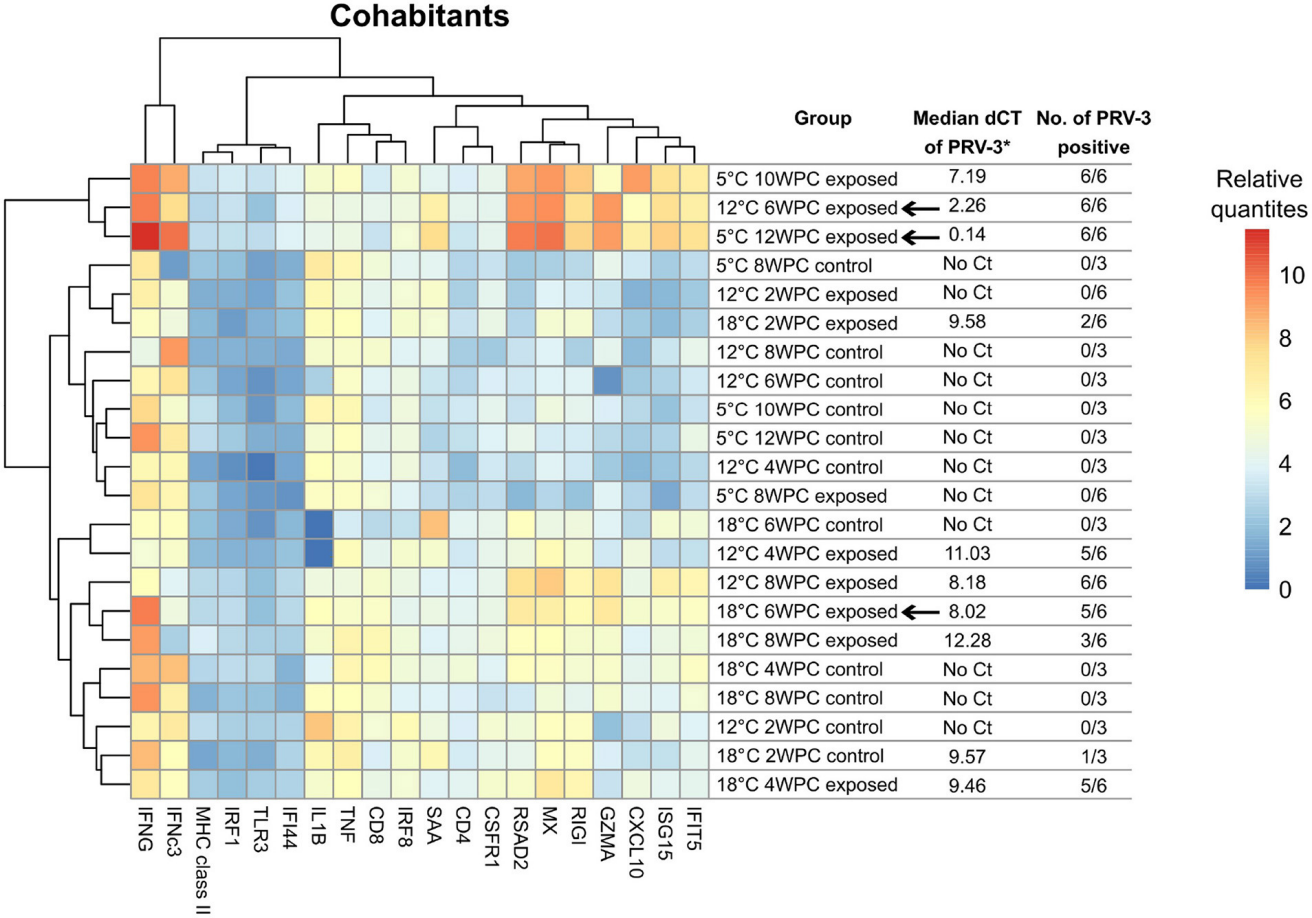
Heatmap of median log2 transformed relative quantities of immune gene expression analyses of selected cohabitant groups. Arrows indicate time point for peak in virus load. ^*^Low value corresponds to high virus load (dCt = Ct[PRV-3] − Ct[Reference gene]). SD values of each group are shown in Supplementary material.

In cohabitant controls, fish maintained at 5 and 12°C showed similar gene expression profiles, while 18°C formed a separate group (see Figure 5). 18°C controls grouped together with PRV-3 exposed fish of the same temperature (with exception of 2 WPC), with only *mhc class II* and *saa* showing significant differences between the control and the exposed fish of 18°C (*p* = 0.003 with fold change of 2.03 and *p* = 0.017 with fold change 0.17, respectively).

Cohabitants control fish at 18°C had up-regulation of *mx, cd4, rsad2, gzma, rigi, isg15, cxcl10, saa* and *ifit5* compared to control fish at 12°C (fold change above 2.5 and *p* < 0.05 in all genes listed).

#### 3.5.2. Temperature induced effect on immune profile of PRV-3 exposed fish

**Figure 6:**
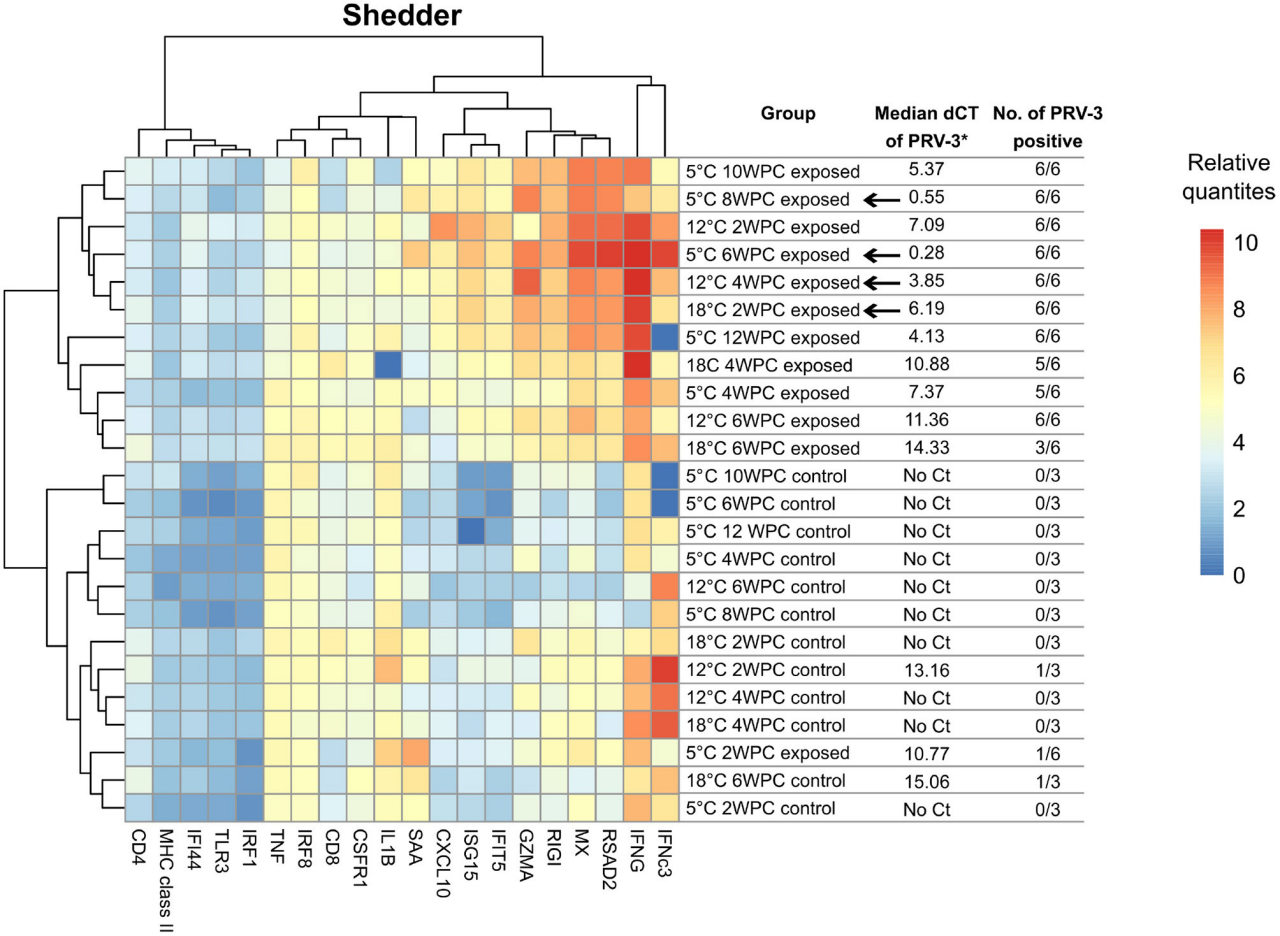
Heatmap of median log2 transformed relative quantities of immune gene expression profiles of selected cohabitant groups. Arrows indicate time point for peak in virus load. ^*^Low value corresponds to high virus load (dCt = Ct[PRV-3] − Ct[Reference gene]). SD values of each group are shown in Supplementary material.

PRV-3 exposed shedder fish of all three temperatures grouped together with the exception of 5°C at 2 WPC (see Figure 6). Across the trial, significant differences were observed at 5°C in *saa* (*p* = 0.002 with fold change 4.6) and 18°C in *cxcl10* (*p* = 0.02 with fold change 0.18) compared to 12°C.

The temperature pattern of *saa* at 5°C changes after viral infection, probably reflecting high viral load and inflammation in the infected spleens at this low temperature.

In cohabitants, fish maintained at 5 and 12°C at the time of highest virus RNA load formed a separate group (10 and 12 WPC at 5°C and 6 WPC at 12°C, see Figure 5). 18°C PRV-3 exposed cohabitants (with the exception of 2 WPC) grouped together with controls of 12°C and 5°C. At the time point of highest virus load at the respective temperatures (6 WPC for both 12 and 18°C, 12 WPC for 5°C), significant differences were observed particularly at 18°C compared to 12°C: here, *mx, rsad2, ifnc3, gzma, rigi, isg15, saa*, and *ifit5* were significantly down-regulated (*p* < 0.05 in all genes), while *cd8* was up-regulated (*p* < 0.05). At 5°C, *ifnc3* and *cxcl10* were up-regulated compared to 12°C at time of high virus load (*p* = 0.0008 with fold change of 4.9 and *p* = 0.018 with fold change of 2.5, respectively).

By comparing the virus load to the immune gene expression of each individual fish, a positive correlation was found in the genes shown in Table 5. Importantly, *isg15* was positively correlated with PRV-3 virus load in all groups, and *rsad2* (viperin) was positively correlated in all groups except 18°C shedders (see Supplementary Figure S4).

**Table 5:** Genes with positive Pearson's correlation to the PRV-3 virus load for all time points with *p* < 0.05.

**Temperature**	**Group**	**Genes**
5°C	Cohabitant	*mx, cd4, ifng, rsad2, irf8, gzma, rigi, isg15, tlr3, cxcl10, saa, ifit5, ifi44, irf1, csf1r*
	Shedder	*mx, rsad2, gzma, rigi, isg15, cxcl10, ifit5, ifi44, irf1*
12°C	Cohabitant	*mx, cd4, rsad2, ifnc3, gzma, rigi, isg15, tlr3, cxcl10, ifit5, ifi44, irf1*
	Shedder	*mx, rsad2, isg15, saa*
18°C	Cohabitant	*ifng, rsad2, gzma, isg15, cxcl10, ifit5, irf1*
	Shedder	*mx, gzma, isg15, cxcl10, ifit5*

## 4. Discussion

RAS farms report to experience severe losses in association with PRV-3 infection, especially during winter and early spring with low water temperature. Hence, in this study, we set out to explore the effect of water temperature on PRV-3 infection in rainbow trout.

In this study, low water temperature (5°C) negatively impacted rainbow trout exposed to PRV-3, inducing a longer incubation period, and yielding higher virus load in shedders with more severe lesions in heart of exposed fish. Shedders at 5°C seemed unable to clear the infection during the course of the experiment: while there was a reduction in virus RNA at 10 weeks post challenge, high virus load persisted until the end of the trial at 12 weeks post challenge. This resembles the observations by Páez et al., showed that long virus persistence and higher long-term virulence was favored at cold water temperature (6°C) in steelhead trout (*Oncorhynchus mykiss*) infected with infectious hematopoietic necrosis virus (IHNV). Complementarily, that study showed that virus persisted for a shorter time and resulted in lower mortality at higher temperatures (10 and 15°C) (24).

Typically, changes in the heart due to PRV infection occur 2 weeks after viral peak under experimental conditions (6, 13). This was delayed in shedder fish at 5°C. Although high virus load (dCt value below 5) was measured starting at 6 WPC, heart lesions were not observed until 10 WPC. However, more severe lesions were found, with half of the fish having the highest histoscore and the other half the second highest histoscore at 12 WPC. Comparing the heart lesions at their respective peak across the three water temperatures (12 WPC at 5°C and 8 WPC at 12°C), there was a tendency toward worse lesions although not significant (*p* = 0.0563).

In cohabitants, low amount of virus was detected until 10 weeks post challenge in the 5°C group, and high virus load was not reached until 12 WPC. Despite the late detection of PRV-3 in this group, there was a significantly higher amount of virus RNA compared to the corresponding time point at 12°C (6 WPC). In contrast to fish at 18°C, fish at 5°C had a reduction of hematocrit at 12 WPC synchronized with the peak in virus RNA load. Heart pathology was not observed in the cohabitants at 5°C, but as changes in the heart are typically observed 2 weeks post highest virus load, it could be expected to show at a later time point which was not covered by the experiment. Additionally, the gene expression profile of the 5°C shedders at 12 WPC differed from the 12°C shedders at 6 WPC: This group had up-regulation of *saa* (*p* = 0.002 with a fold change at 4.6), and down-regulation of *cd8* and *cxcl10* (*p* < 0.001 and *p* = 0.002 at fold change 0.4 and 0.3, respectively).

The PRV-3 exposed shedder fish at 5°C at 2 WPC had immune gene expression profile more similar to the controls than the other exposed fish (see Figure 6). It seems that, despite the i.p. injection of PRV-3 positive inoculum, it took longer time to trigger the immune response due to the low water temperature.

Cohabitants at 10 and 12 weeks post challenge (high virus RNA load) at 5°C had a similar gene expression profile as fish in 12°C water at the corresponding time point (6 weeks post challenge), with up-regulation of *ifng*. However, comparing 5°C at 12 WPC to 12°C at 6 WPC, a significant up-regulation of *ifnc3* and *cxcl10* was observed at 5°C.

The protective effect of interferon C has already been shown in Atlantic salmon challenged with ISAV. By injecting *ifnc* encoding plasmid along with ISAV vaccine, it was possible to highlight the role of IFN in promoting ISG expression and boosting specific protection and antibody response (35, 36). Similarly, Zhang et al. (37) have shown that early activation of interferon pathway prevent second replication of the virus using as experimental model fish hirame rhabdovirus in flounders, a similar effect has also been shown for a number of fish viral infections [e.g., VHSV, IHNV, and RGNNV (red grouper nervous necrosis virus)] (38–41). In our experiment, we report significant overexpression of *ifnc3* in shedder fish at 12 and 18°C compared to 5°C (*p* = 6.1E-05 and *p* = 0.0132, respectively), when grouping together all time points. *ifnc3* overexpression likely limited the replication of PRV-3, preventing further shedding, and heart pathology development. Additionally, *cxcl10* is highly expressed in a wide range of tissues following viral infection, including influenza A virus and SARS-CoV-2 (42, 43). Expression of *cxcl10* is stimulated by *IFN* and involved in inflammation and chemotaxis of lymphocytes including T lymphocytes and natural killer cells during viral infections.

*cxcl10* is an important antiviral gene and is highly correlated with viral load in the present study (Table 5). *cxcl10* is expressed in a wide range of tissues following viral infection, including influenza A virus and SARS-CoV-2 (42, 43). Expression of *cxcl10* is stimulated by IFN and involved in inflammation and chemotaxis of lymphocytes including T lymphocytes and NK cells during viral infections. Thus, the high level of this anti-viral chemokine at 5°C might be explained by the high viral load found at the same temperature. In agreement with exposed shedders, cohabitant fish maintained at 5°C show a high expression of the ISG *cxcl10*, reflecting the high viral load. Type 1 interferon expression (*ifnc3*) was likewise found to be increased at low temperatures. *ifnc* has previously been shown to protect against Salmonid alphavirus 3 (SAV3), probably by inducing expression of the ISG *mx* also found to be correlated with viral titers in the present study (44).

Shedder control fish at 5°C showed down-regulation of *ifnc3* compared to 12°C group. The significant down-regulation of ifnc3 at 5°C could be a result of low levels of the pattern recognition receptor (PRR) *rigi* also found at 5°C. *rigi* is central in activating a wide range of IFNs after nucleic acid recognition from invading viral pathogens. Low levels of IFN including *ifnc3* will results in compromised activation of important interferon stimulated genes (ISG) including *isg15, ifit5* and *ifi44*. The combined action of these and other ISG are crucial in control and disease outcome after viral infections. Importantly, reduced expression of ISG at 5°C might affect the course and outcome of subsequent viral infections including PRV-3. Significant upregulation of the acute-phase protein (APP) saa at 18°C was also seen in the mock-injected shedders. APPs are normally produced in the liver and rises as a response to inflammation and tissue injury. Extra-hepatic changes of APPs upon viral infection has previously been reported during influenza A virus infection in pigs (34), but the link between saa, inflammation and water temperature still needs to be elucidated.

In this experiment, we observe little to no regulation of both *cd4* and *cd8*. In a previous experiment with PRV-3, these two genes have been up-regulated in relation to heart pathology (13). However, it is important to note that in the aforementioned study, immune gene expression was performed on heart tissue, while we here looked at the spleen. This may explain the lack of regulation of *cd4* and *cd8*.

Importantly, *isg15* was shown to be positively correlated with PRV-3 virus load in all PRV-3 exposed groups, while *rsad2* (viperin) and *mx* were positively correlated with PRV-3 virus load in all groups with exception of 18°C shedders and 18°C cohabitants, respectively (see Supplementary Tables S2–S14 and Supplementary Figure S4).

Rainbow trout are poikilothermic animals, which has significant effect on the kinetics of a large number of metabolic processes including immune response. To normalize the effect of temperature, we explored the data of viral load as function of degree days (Supplementary Figure S1). Overall, this approach shows an interesting alignment in degree days for reaching viral peak in shedders at 5 and 12°C. High PRV-3 virus load is detected earlier at 5°C than at 12 and 18°C in both cohabitants and shedders (see Supplementary Figure S1). This is likely to reflect slight changes in sampling point. Notably, worse heart pathology was observed at 5°C shedders at peak of infection.

In fish, the phenomenon of behavioral fever has been studied as a response to infections, particularly to viruses (25). Behavioral fever induces the fish to shift from optimal temperature moving to a warmer area with increased oxygen consumption and reduction of oxygen dissolved in the water. The increase of environmental temperature has a double effect both boosting the immune response and modulating the replication capacity of the pathogen (26). Induction of behavioral fever has been shown in zebra fish exposed to dsRNA (45). With regards to PRV infection in salmonids, it is reported that HSMI outbreaks tend to appear more frequently late spring-early summer (27), and it is documented that HSMI affected fish in sea cage are close to the surface (28). Similarly, rainbow trout suffer severe disease outbreak during winter, and during the outbreak tend to swim in close proximity of the surface, possibly searching for the warmest temperature available in the confined environment to develop a behavioral fever. Recent studies by Boltana and colleagues (29) highlight how behavioral fever modulates epigenetic expression of immune response, showing how Atlantic salmon exposed to another double stranded RNA virus (IPNV); by shifting to higher water temperature fish activates transcription of a specific subset of mRNA for innate and adaptive immune response, potentiating its mitigating effect on infection.

In our experiment, high water temperature (18°C) had a positive effect in mitigating the infection. At 18°C water temperature, both shedders and cohabitants had a tendency toward lower virus RNA load compared to 12°C (AUC, *p* < 0.0001). Although we demonstrated some PRV-3 replication, the reduction in hematocrit, which is normally observed at the same time point as peak in virus RNA, did not occur. Strikingly, these fish (18°C shedders and cohabitants) did not develop any heart pathology either. Cohabitants maintained at 18°C had an up-regulation of *cd8*, and down-regulation of immune genes of the innate response (*mx, rsad2, ifnc3, gzma, rigi, isg15*, and *saa*) compared to 12°C at 6 weeks post challenge (*p* < 0.05 in all genes) which mitigated the negative effect of PRV-3 infection in rainbow trout. Further studies will focus on how water temperature control could be practically implemented in future RAS farm design for mitigation of infectious diseases.

Taking into consideration the viral kinetics and heart pathology development both at 12 and 18°C, it appears that a threshold in virus load is required in order to trigger the development of heart pathology in rainbow trout due to PRV-3 infection. In shedder fish at 12°C, but not at 18°C, heart lesions were observed following high virus load. Here, a dCt value below 5 dCt at 12°C resulted in heart lesions, while a dCt value above 5 in 18°C did not trigger heart pathology. The same case can be observed in the cohabitants at 12 and 18°C; a dCt value above 5 did not result in histopathology in contrast to a dCt value below 5 (Figures 4G, H, I). Additionally, *rigi* appear to be a possible immune marker predictive of heart histopathology for PRV-3 infection. Within the pool of *rigi, ifit5*, and *rsad2*, this gene was highly expressed 2–4 weeks prior to the occurrence of lesions in the heart of both shedder and cohabitant fish at 5 and 12°C, but not at 18°C (see Supplementary Figure S2). In a study using human cardiac fibroblasts, *rigi* has been shown to be involved in cardiac cell pathology, as expression of *rigi* promoted production of pro-inflammatory cytokines which contributed to heart injury (46).

Fish were not acclimatized before the exposure to PRV-3, thus it is important to consider the potential impact of the sudden temperature shift on the fish. In cohabitants, controls of 5 and 12°C had a relatively similar gene expression profile, while controls of 18°C formed a separate group (see Figure 5), primarily driven by the deferentially expression of *mx, cd4, rsad2, gzma, rigi, isg15, cxcl10, saa*, and *ifit5* (up-regulated in controls of 18°C compared to 12°C). However, these differences persisted throughout the trial, and should therefore not be attributed to the lack of acclimatization, but rather an impact of the temperature on the base level of immune gene expression. In shedders, all controls showed similar gene profile. In the shedder control fish of 12°C two and four WPC and 18°C four WPC, high expression of interferon's was observed. This short increase in interferon expression could be a reaction to the mock-injection. Nevertheless, the expression of both *ifng* and *ifnc3* decreased after some time.

In conclusion, low water temperature negatively impacts PRV-3 exposed fish, as virus replication persists for longer compared to higher temperatures. Additionally, heart pathology was indicated to be worsened by low temperature, while high temperature negates the negative impact of PRV-3 infection. It could be speculated, if the movement of at risk fish batches into warm water compartments could prevent disease outbreaks caused by PRV-3.

## Data availability statement

The original contributions presented in the study are included in the article/Supplementary material, further inquiries can be directed to the corresponding author.

## Ethics statement

The animal study was reviewed and approved by the Animal Experiments Inspectorate (Ministry of Food, Agriculture and Fisheries of Denmark).

## Author contributions

NV, NO, AC, and JS contributed to the conceptualization and design of the study. Methodology, data analysis, and visualization done by JS, AC, NV, AO, and KS. Data curation done by JS and AO. Statistical analyses and writing of original draft done by JS. Review and editing done by JS, NV, NO, AC, AO, TI, and KS. Project administration done by NV and NO. Funding acquisition done by NV. All authors have read and agree to the published version of the manuscript.
