# Supplementary material for: Decreased water temperature enhance Piscine orthoreovirus genotype 3 replication and severe heart pathology in experimentally infected rainbow trout
Source: Front Vet Sci. 2023 Feb 10;10:1112466. doi: 10.3389/fvets.2023.1112466 (PMC9950551; doi:10.3389/fvets.2023.1112466)
Supplement: Supplementary file 1 [file Data_Sheet_1.pdf]

Supplementary Material

1 SUPPLEMENTARY TABLES AND FIGURES

Table S1. PRV-3 artificial control

Sequence of PRV-3 gBlock
TACAGGTCGTGTTCCCGTTGTGGTGTGGGA
GGAACCGAGAGAAGAATACAGATTCAGGTT
GGATGGAGCGCGTGATCTACCTCGTGGCTG
GAAAAACGATCTTCAAGGGGACTGAAGAC
AACAGTCGTGGTTCCAATGACAGACCAGAC
CGACGGGGGATCGCTGAAGCTAAGCGACG
CCTTAGAGACAACATGCGAAGCATAATGAA
GAAAAAGACCACTGGTGATCTTGGTTTTA
GTGGTTGGATGGTCCTCGACCCGGA

Table S2. Standard deviation (SD) of immune gene expression log2 transformed RQ values for each experimental group. NA indicated that there was only one individual in the group, and therefore SD could not be calculated.

Temperature	Group	Treatment	WPC	mx	cd4	cd8	ifng	rsad2	ifnc3	tnf	il1b	irf8	mhc class II	gzma	RIG-I	isg15	tlr3	cxcl10	saa	ifit5	ifi44	irf1	csf1r
5	Cohabitant	Control	8	2.093296	0.034765	0.097156	2.255975	0.970114	1.548104	0.859753	2.343717	0.250795	1.367751	0.039359	0.621084	0.321903	0.120263	0.332733	2.554999	0.723127	0.384918	0.321055	1.010542
5	Cohabitant	Control	10	0.573663	0.390991	0.97405	3.581744	0.438219	1.633648	0.504056	0.268426	0.097538	0.310938	0.55411	0.444786	0.420311	0.101521	0.326697	0.286345	0.324196	0.48271	0.1076	0.210913
5	Cohabitant	Control	12	0.922571	0.366948	0.553058	3.545259	1.895405	4.993577	0.359538	0.067698	0.445683	0.887701	0.334683	1.147686	0.156627	0.594047	0.906616	1.206333	0.775834	1.068863	0.083014	0.564942
12	Cohabitant	Control	2	0.509973	0.330912	0.164918	2.27136	0.472353	5.265321	0.301757	0.992578	0.193232	0.231128	0.998991	0.393791	0.348959	0.174764	0.761034	1.437046	0.375091	0.08654	0.180291	0.306805
12	Cohabitant	Control	2	NA	NA	NA	NA	NA	NA	NA	NA	NA	NA	NA	NA	NA	NA	NA	NA	NA	NA	NA	NA
12	Cohabitant	Control	4	0.519012	0.591507	1.175392	1.353139	0.63464	1.711373	1.171978	0.430364	1.247913	0.245875	0.82962	0.447382	0.362198	0.339652	0.644101	0.634196	0.406908	0.238362	0.522818	0.572031
12	Cohabitant	Control	6	0.262861	0.000718	0.768354	4.885569	0.972434	2.802414	0.255236	3.670766	1.256863	0.092072	1.282646	0.051516	1.907916	0.166386	1.449058	1.539455	1.254684	0.003762	1.073429	0.937233
12	Cohabitant	Control	8	1.149551	0.726911	0.278427	6.29767	0.699172	4.281492	0.001522	0.652981	0.222555	0.156302	0.966933	0.359759	0.863403	0.560868	0.009892	0.908829	0.649811	0.650707	0.350589	0.285149
18	Cohabitant	Control	2	0.274681	0.35029	0.793227	1.495871	0.722867	0.434274	2.548139	2.97925	0.185154	0.445403	0.593421	0.257353	0.357077	0.612214	0.752613	1.829564	0.534552	0.188725	0.533259	0.673007
18	Cohabitant	Control	4	0.144732	0.818828	3.304696	0.671737	0.915796	8.545046	0.253895	5.389092	2.306028	0.425599	0.413226	0.186172	2.32575	1.288857	1.359642	1.565257	3.494625	1.009111	1.655993	2.269074
18	Cohabitant	Control	6	1.45988	1.50943	1.991688	5.172099	1.429277	1.401997	3.780961	6.780622	1.07676	0.261365	1.551579	2.005832	1.006501	0.18648	1.003802	2.543231	1.083551	0.528436	1.458434	1.275201
18	Cohabitant	Control	8	0.309014	0.1212	0.724183	1.097629	1.267348	0.915339	0.456489	0.502991	0.627176	0.66189	1.212861	0.619023	0.880184	0.42577	1.171898	0.871052	0.332454	0.216857	0.275687	0.619859
5	Cohabitant	Exposed	8	0.671407	0.207799	0.847833	3.308122	0.937155	3.092958	1.874277	0.873754	0.428742	0.43318	0.81504	0.691722	0.906648	0.563549	0.750958	1.134484	1.09865	0.412456	0.381107	0.592061
5	Cohabitant	Exposed	10	2.005426	0.5667	0.809516	3.703872	2.050278	1.453628	0.403289	0.859595	0.462203	0.55295	1.135726	1.663785	2.144937	0.876416	2.502419	0.927329	1.406573	0.901668	1.063971	0.367956
5	Cohabitant	Exposed	12	0.45377	0.496047	0.487796	2.562416	0.582998	0.910111	0.748918	1.943657	0.530591	0.877735	0.444289	0.576164	0.325959	0.481365	0.751209	0.157633	0.388665	0.439782	0.438096	0.404099
12	Cohabitant	Exposed	2	0.850146	0.222247	0.512072	1.517388	0.747241	3.401544	1.457958	0.722954	0.327523	0.267295	1.773597	0.865497	1.366728	0.600256	0.817466	1.646339	0.633425	0.581714	0.387505	0.310619
12	Cohabitant	Exposed	4	3.166662	0.336294	1.20948	5.580979	2.995532	4.423533	0.766015	3.482712	1.858498	0.441625	2.758097	1.290968	3.041499	0.455473	2.657085	1.061876	2.662297	0.576772	1.071714	1.575449
12	Cohabitant	Exposed	6	0.318237	0.422171	1.089761	3.185683	0.75489	0.738546	0.993069	2.970164	0.773743	0.703379	0.538914	0.732862	0.42465	0.6412	0.620825	0.996908	0.428068	0.319354	0.383902	0.817996
12	Cohabitant	Exposed	8	0.66351	0.533549	0.293591	4.349853	0.64385	2.423148	0.381363	0.604832	0.557122	0.775334	0.490257	0.363159	0.716664	0.207251	0.778458	1.227045	0.815132	0.336238	0.240599	0.599865
18	Cohabitant	Exposed	2	1.346406	0.263339	0.454615	2.599429	0.842157	0.737027	0.437668	3.778455	0.364849	0.689833	0.265569	1.098979	0.69861	0.590069	0.562743	1.648906	0.67952	0.503111	0.122745	0.383678
18	Cohabitant	Exposed	4	1.293778	0.112034	0.59201	3.158555	2.51711	3.269891	0.845532	2.206207	0.761727	0.211469	1.575781	1.295176	2.373447	0.64066	2.008267	0.434934	1.692846	0.70415	0.921542	0.744435
18	Cohabitant	Exposed	6	1.177242	0.387614	0.769983	2.677416	1.149207	2.159056	1.110643	0.77565	0.655338	0.640728	1.303309	0.786646	0.446464	0.706613	0.765007	1.282595	0.26211	0.419899	0.376697	0.267445
18	Cohabitant	Exposed	8	0.757533	0.479923	0.610895	0.264998	0.448779	3.770979	1.071403	1.249056	0.455188	0.443118	1.198307	0.568617	0.698126	0.568865	0.874543	1.258305	0.800436	0.451502	0.373974	0.647302
5	Shedder	Control	2	1.8656	0.125752	0.391615	0.600819	1.60935	0.585841	0.161716	0.511579	0.627891	0.352516	0.033941	1.539175	1.509629	0.606449	0.465909	0.435759	1.315885	0.476065	0.270095	0.395011
5	Shedder	Control	4	0.485549	0.571032	0.266889	0.052194	1.040015	0.942052	0.269711	0.630573	0.245278	0.481665	0.772298	0.182865	1.911488	0.013007	0.195823	0.195081	0.699378	0.160183	0.030159	0.695009
5	Shedder	Control	6	0.387599	0.280218	0.33222	2.524824	0.491335	3.192066	0.69168	0.289167	0.389882	0.4501	1.092336	0.636171	1.159116	0.445358	1.323702	0.420836	1.432467	0.162773	0.805748	0.5805
5	Shedder	Control	8	0.575247	0.260282	0.473119	3.60023	0.601985	4.235619	0.455807	0.167664	0.462249	0.559039	0.952407	0.595468	0.770671	0.224387	0.732188	0.663577	0.429992	0.491224	0.408037	0.309948
5	Shedder	Control	10	0.916559	0.675657	0.288785	1.623171	0.367888	3.618001	0.087168	0.369268	0.899535	1.320613	0.824044	0.984561	0.020346	0.145489	0.581455	1.234747	0.46473	0.632426	0.61266	0.677425
5	Shedder	Control	12	0.167234	0.076109	0.642197	0.478498	0.558429	1.001837	0.246672	0.434603	0.387939	0.517285	0.662832	0.735973	1.007528	0.192858	0.68003	0.777674	0.932094	0.286932	0.450166	0.397523
12	Shedder	Control	2	0.570056	0.324067	0.296321	0.687709	0.993928	0.342647	0.05963	1.432504	0.069579	0.329622	2.087451	0.236967	0.447999	0.124853	0.450528	1.307731	0.232004	0.106792	0.096381	0.321425
12	Shedder	Control	4	1.289694	0.27642	0.662003	2.519896	0.92292	1.44107	0.543361	3.112812	0.367664	0.238908	2.291941	0.818636	0.435587	0.481313	0.769468	1.071477	0.691909	0.424287	0.221545	0.262868
12	Shedder	Control	6	1.927192	0.278032	0.835713	4.017043	0.646994	3.747923	1.063674	3.112533	0.499628	0.986906	1.429975	2.542687	0.178999	0.563602	0.116188	0.948268	0.913533	0.454384	0.241845	1.094924
18	Shedder	Control	4	0.857313	0.151013	0.528071	5.486052	1.07862	1.635947	0.518353	0.915792	0.312355	0.431562	1.223221	0.689986	1.334508	0.818167	0.635495	0.330257	0.997481	0.553067	0.401034	0.463232
18	Shedder	Control	6	1.832841	0.359431	2.568354	2.299388	0.666375	5.023589	1.787147	3.859378	0.047639	0.439959	1.016622	0.773967	0.919237	0.687434	0.974778	2.743783	1.260031	0.227595	1.001887	0.385138
5	Shedder	Exposed	2	2.196675	0.68134	0.921462	3.983518	2.720342	3.22689	1.244085	0.989627	0.3161	0.738614	0.764802	1.393454	2.336618	0.629766	0.542292	1.777979	1.896355	0.740686	0.289171	0.630357
5	Shedder	Exposed	4	1.182368	0.237114	0.455567	4.288885	1.925351	1.897821	0.764411	0.873018	0.342536	0.315104	0.721783	1.143753	2.03087	0.516228	1.884085	1.461955	1.369035	0.710204	0.646227	0.424183
5	Shedder	Exposed	6	0.545178	0.390289	0.669956	4.566992	0.310206	1.642906	0.6754	0.568553	0.614961	0.539686	0.702417	0.603868	0.759785	0.484442	0.661698	0.989524	0.569917	0.521417	0.259555	0.580309
5	Shedder	Exposed	8	1.636738	0.402517	0.788152	3.828166	2.826603	1.35003	1.033771	2.078022	0.515572	0.287275	2.261052	1.587309	2.376392	0.555925	0.852502	2.077855	2.08198	0.872268	0.229705	0.385402
5	Shedder	Exposed	10	0.523265	0.304922	0.731972	3.185629	0.582922	2.701561	1.729797	3.061577	0.188885	0.45278	0.974023	0.445579	0.353717	0.49473	0.358074	1.313012	0.575538	0.267543	0.390004	0.259858
5	Shedder	Exposed	12	0.667599	0.313722	0.556749	0.375258	0.60499	2.819257	0.66879	0.932923	0.283176	0.769373	0.541338	0.302302	0.291996	0.352195	0.390447	1.675241	0.363939	0.233017	0.237891	0.41954
12	Shedder	Exposed	2	0.622615	0.446787	0.223132	3.426648	0.518792	1.781457	0.692393	1.093064	0.284991	0.54657	0.994522	0.423689	0.529233	0.228664	0.693218	1.10354	0.5113	0.240705	0.321032	0.302844
12	Shedder	Exposed	4	0.655809	0.293435	0.237397	0.443184	0.357912	1.919098	0.851418	2.566566	0.227915	0.456376	0.498731	0.736214	0.275738	0.309176	0.534534	0.848464	0.315483	0.614269	0.244146	0.5501
12	Shedder	Exposed	6	0.595175	0.456533	0.294301	2.794087	0.539812	2.690358	0.626689	2.693643	0.373167	0.593218	0.28561	0.537864	0.488434	0.31572	0.924542	0.37076	0.276838	0.175245	0.201584	0.390492
18	Shedder	Exposed	2	0.37131	0.398008	0.646227	4.038654	0.582411	0.724411	0.887037	2.421105	0.499143	0.327086	0.804779	0.561	0.510218	0.705125	0.900258	1.33904	0.325588	0.584901	0.601683	0.714002
18	Shedder	Exposed	4	1.329644	0.356909	0.158962	2.621194	1.108877	4.034888	2.101036	2.099116	0.325008	0.762527	1.124237	1.322044	0.2296	0.342355	0.771685	0.808744	0.201313	0.497511	0.278357	0.657842
18	Shedder	Exposed	6	0.639557	0.332448	0.656493	2.384753	0.439294	2.222889	0.80441	2.697422	0.421576	0.578338	1.077883	0.347431	0.51679	0.262574	0.358344	0.989225	0.656758	0.317698	0.432788	0.484799

Table S3. Pearson's correlation analysis of 5°C PRV-3 exposed cohabitants (based on all time points). *As dCt is calculated as Ct[PRV-3]-Ct[Reference gene], the lowest dCt value corresponds to the highest virus load. Therefore, a negative value in the correlation analysis for PRV-3 dCt is a positive correlation.

	mx	*cd4*	*cd8*	*ifng*	*rsad2*	*ifnc3*	*tnf*	*il1b*	*irf8*	*mhc class II*	*gzma*	*rigi*	*isg15*	*tlr3*	*cxcl10*	*saa*	*ifit5*	*ifi44*	*irf1*	*csf1r*	**PRV3 dCt**
mx	1.00	0.72	-0.79	0.61	0.99	0.69	0.14	-0.33	0.76	0.62	0.74	0.98	0.98	0.90	0.88	0.72	0.96	0.95	0.91	0.67	-0.83
cd4	0.72	1.00	-0.63	0.63	0.72	0.53	0.01	-0.04	0.68	0.61	0.58	0.72	0.70	0.67	0.72	0.41	0.71	0.73	0.78	0.66	-0.56
cd8	-0.79	-0.63	1.00	-0.58	-0.81	-0.58	-0.10	0.22	-0.76	-0.68	-0.60	-0.83	-0.81	-0.80	-0.74	-0.55	-0.70	-0.78	-0.74	-0.76	0.65
ifng	0.61	0.63	-0.58	1.00	0.64	0.32	0.44	-0.18	0.39	0.42	0.46	0.61	0.60	0.35	0.56	0.44	0.54	0.57	0.58	0.49	-0.47
rsad2	0.99	0.72	-0.81	0.64	1.00	0.72	0.16	-0.37	0.77	0.61	0.77	0.98	0.99	0.89	0.88	0.75	0.95	0.96	0.91	0.70	-0.85
ifnc3	0.69	0.53	-0.58	0.32	0.72	1.00	-0.18	-0.17	0.68	0.55	0.76	0.69	0.71	0.76	0.65	0.61	0.72	0.74	0.75	0.46	-0.70
tnf	0.14	0.01	-0.10	0.44	0.16	-0.18	1.00	0.33	0.08	-0.07	-0.26	0.18	0.14	-0.04	0.27	0.05	0.05	0.12	0.15	0.14	0.07
il1b	-0.33	-0.04	0.22	-0.18	-0.37	-0.17	0.33	1.00	-0.19	0.01	-0.44	-0.32	-0.33	-0.13	-0.09	-0.45	-0.30	-0.34	-0.13	-0.32	0.44
irf8	0.76	0.68	-0.76	0.39	0.77	0.68	0.08	-0.19	1.00	0.68	0.45	0.83	0.75	0.82	0.84	0.38	0.71	0.86	0.76	0.81	-0.56
mhc class II	0.62	0.61	-0.68	0.42	0.61	0.55	-0.07	0.01	0.68	1.00	0.36	0.64	0.61	0.71	0.71	0.16	0.64	0.65	0.68	0.62	-0.39
gzma	0.74	0.58	-0.60	0.46	0.77	0.76	-0.26	-0.44	0.45	0.36	1.00	0.69	0.79	0.69	0.49	0.85	0.75	0.68	0.65	0.40	-0.90
rigi	0.98	0.72	-0.83	0.61	0.98	0.69	0.18	-0.32	0.83	0.64	0.69	1.00	0.98	0.90	0.92	0.68	0.94	0.98	0.92	0.74	-0.78
isg15	0.98	0.70	-0.81	0.60	0.99	0.71	0.14	-0.33	0.75	0.61	0.79	0.98	1.00	0.92	0.87	0.73	0.96	0.95	0.93	0.63	-0.83
tlr3	0.90	0.67	-0.80	0.35	0.89	0.76	-0.04	-0.13	0.82	0.71	0.69	0.90	0.92	1.00	0.86	0.56	0.90	0.88	0.89	0.60	-0.73
cxcl10	0.88	0.72	-0.74	0.56	0.88	0.65	0.27	-0.09	0.84	0.71	0.49	0.92	0.87	0.86	1.00	0.45	0.86	0.93	0.93	0.70	-0.55
saa	0.72	0.41	-0.55	0.44	0.75	0.61	0.05	-0.45	0.38	0.16	0.85	0.68	0.73	0.56	0.45	1.00	0.63	0.65	0.54	0.42	-0.87
ifit5	0.96	0.71	-0.70	0.54	0.95	0.72	0.05	-0.30	0.71	0.64	0.75	0.94	0.96	0.90	0.86	0.63	1.00	0.93	0.94	0.57	-0.80
ifi44	0.95	0.73	-0.78	0.57	0.96	0.74	0.12	-0.34	0.86	0.65	0.68	0.98	0.95	0.88	0.93	0.65	0.93	1.00	0.92	0.74	-0.75
irf1	0.91	0.78	-0.74	0.58	0.91	0.75	0.15	-0.13	0.76	0.68	0.65	0.92	0.93	0.89	0.93	0.54	0.94	0.92	1.00	0.58	-0.66
csf1r	0.67	0.66	-0.76	0.49	0.70	0.46	0.14	-0.32	0.81	0.62	0.40	0.74	0.63	0.60	0.70	0.42	0.57	0.74	0.58	1.00	-0.57
**PRV3 dCt**	-0.83	-0.56	0.65	-0.47	-0.85	-0.70	0.07	0.44	-0.56	-0.39	-0.90	-0.78	-0.83	-0.73	-0.55	-0.87	-0.80	-0.75	-0.66	-0.57	1.00

Table S4. P-value for Pearson's correlation analysis of 5°C PRV-3 exposed cohabitants (based on all time points).

	mx	***cd4***	***cd8***	***ifng***	***rsad2***	***ifnc3***	***tnf***	***il1b***	***irf8***	***mhc class II***
mx	0	0.000853	8.44E-05	0.007642	0	0.001397	0.585122	0.175232	0.000262	0.005572
cd4	0.000853	0	0.00519	0.00517	0.000753	0.024223	0.956806	0.878964	0.002078	0.007262
cd8	8.44E-05	0.00519	0	0.011409	5.41E-05	0.010929	0.687024	0.383859	0.00029	0.001872
ifng	0.007642	0.00517	0.011409	0	0.004452	0.200607	0.070658	0.466764	0.107359	0.084929
rsad2	0	0.000753	5.41E-05	0.004452	0	0.000724	0.534976	0.136382	0.000168	0.007696
ifnc3	0.001397	0.024223	0.010929	0.200607	0.000724	0	0.462448	0.500831	0.002048	0.016842
tnf	0.585122	0.956806	0.687024	0.070658	0.534976	0.462448	0	0.186562	0.750981	0.782932
il1b	0.175232	0.878964	0.383859	0.466764	0.136382	0.500831	0.186562	0	0.449057	0.968073
irf8	0.000262	0.002078	0.00029	0.107359	0.000168	0.002048	0.750981	0.449057	0	0.002083
mhc class II	0.005572	0.007262	0.001872	0.084929	0.007696	0.016842	0.782932	0.968073	0.002083	0
gzma	0.000427	0.012343	0.00869	0.057197	0.000204	0.000292	0.306514	0.06453	0.064017	0.141311
rigi	0	0.000726	1.95E-05	0.007492	0	0.0016	0.462801	0.188522	1.84E-05	0.004023
isg15	0	0.001276	4.65E-05	0.008754	0	0.000855	0.587996	0.185764	0.000364	0.006694
tlr3	3E-07	0.002583	7.17E-05	0.159965	9.9E-07	0.000255	0.882262	0.602792	3.72E-05	0.000871
cxcl10	1.12E-06	0.000797	0.000464	0.016493	1.34E-06	0.00363	0.278654	0.723526	1.49E-05	0.000922
saa	0.000799	0.087946	0.018416	0.071188	0.000302	0.007643	0.84755	0.059284	0.116892	0.537285
ifit5	0	0.000881	0.001146	0.021553	0	0.000795	0.836113	0.227033	0.00089	0.003982
ifi44	0	0.000545	0.000127	0.014387	0	0.000471	0.633536	0.173306	5.62E-06	0.003419
irf1	1.1E-07	0.000117	0.0005	0.011228	1.1E-07	0.000363	0.556669	0.608651	0.000249	0.001919
csf1r	0.002492	0.003035	0.000261	0.039721	0.0012	0.056301	0.566851	0.202211	4.27E-05	0.005946
**PRV3 dCt**	1.84E-05	0.014831	0.003412	0.04902	9.15E-06	0.001348	0.783142	0.064673	0.016205	0.106912

	gzma	***rigi***	***isg15***	***tlr3***	***cxcl10***	***saa***	***ifit5***	***ifi44***	***irf1***	***csf1r***	**PRV3 dCt**
mx	0.000427	0	0	3E-07	1.12E-06	0.000799	0	0	1.1E-07	0.002492	1.84E-05
cd4	0.012343	0.000726	0.001276	0.002583	0.000797	0.087946	0.000881	0.000545	0.000117	0.003035	0.014831
cd8	0.00869	1.95E-05	4.65E-05	7.17E-05	0.000464	0.018416	0.001146	0.000127	0.0005	0.000261	0.003412
ifng	0.057197	0.007492	0.008754	0.159965	0.016493	0.071188	0.021553	0.014387	0.011228	0.039721	0.04902
rsad2	0.000204	0	0	9.9E-07	1.34E-06	0.000302	0	0	1.1E-07	0.0012	9.15E-06
ifnc3	0.000292	0.0016	0.000855	0.000255	0.00363	0.007643	0.000795	0.000471	0.000363	0.056301	0.001348
tnf	0.306514	0.462801	0.587996	0.882262	0.278654	0.84755	0.836113	0.633536	0.556669	0.566851	0.783142
il1b	0.06453	0.188522	0.185764	0.602792	0.723526	0.059284	0.227033	0.173306	0.608651	0.202211	0.064673
irf8	0.064017	1.84E-05	0.000364	3.72E-05	1.49E-05	0.116892	0.00089	5.62E-06	0.000249	4.27E-05	0.016205
mhc class II	0.141311	0.004023	0.006694	0.000871	0.000922	0.537285	0.003982	0.003419	0.001919	0.005946	0.106912
gzma	0	0.001552	0.0001	0.001602	0.040712	7.25E-06	0.000385	0.001882	0.003203	0.104306	3.8E-07
rigi	0.001552	0	0	3E-07	8E-08	0.002032	1E-08	0	7E-08	0.000459	0.000115
isg15	0.0001	0	0	9E-08	2.14E-06	0.000526	0	0	3E-08	0.004696	1.68E-05
tlr3	0.001602	3E-07	9E-08	0	4.59E-06	0.015835	3E-07	1.43E-06	6.3E-07	0.00805	0.000557
cxcl10	0.040712	8E-08	2.14E-06	4.59E-06	0	0.060816	5.23E-06	2E-08	4E-08	0.001216	0.017827
saa	7.25E-06	0.002032	0.000526	0.015835	0.060816	0	0.005457	0.003258	0.022074	0.078827	2.89E-06
ifit5	0.000385	1E-08	0	3E-07	5.23E-06	0.005457	0	2E-08	0	0.014202	7.92E-05
ifi44	0.001882	0	0	1.43E-06	2E-08	0.003258	2E-08	0	8E-08	0.000448	0.000357
irf1	0.003203	7E-08	3E-08	6.3E-07	4E-08	0.022074	0	8E-08	0	0.01111	0.002927
csf1r	0.104306	0.000459	0.004696	0.00805	0.001216	0.078827	0.014202	0.000448	0.01111	0	0.013833
**PRV3 dCt**	3.8E-07	0.000115	1.68E-05	0.000557	0.017827	2.89E-06	7.92E-05	0.000357	0.002927	0.013833	0

Table S5. Pearson's correlation analysis of 5°C PRV-3 exposed shedders (based on all time points). *As dCt is calculated as Ct[PRV-3]-Ct[Reference gene], the lowest dCt value corresponds to the highest virus load. Therefore, a negative value in the correlation analysis for PRV-3 dCt is a positive correlation.

	mx	*cd4*	*cd8*	*ifng*	*rsad2*	*ifnc3*	*tnf*	*il1b*	*irf8*	*mhc class II*	*gzma*	*rigi*	*isg15*	*tlr3*	*cxcl10*	*saa*	*ifit5*	*ifi44*	*irf1*	*csf1r*	**PRV3 dCt**
mx	1	0.559276	-0.19223	0.249919	0.96307	0.255269	-0.70112	-0.47598	0.068768	0.455564	0.843668	0.946853	0.947097	0.693558	0.636009	0.266564	0.934003	0.931619	0.676523	0.209491	-0.68181
cd4	0.559276	1	-0.35004	-0.06948	0.618414	0.025064	-0.50137	-0.21989	0.28344	0.510124	0.584331	0.678008	0.521577	0.481249	0.073356	0.253422	0.511824	0.62153	0.221622	0.529806	-0.24201
cd8	-0.19223	-0.35004	1	0.110959	-0.18158	-0.00284	0.253285	0.165172	-0.20286	-0.11641	-0.10303	-0.29327	-0.0824	-0.07423	0.226955	-0.24585	-0.07371	-0.2532	0.1386	-0.07668	0.044936
ifng	0.249919	-0.06948	0.110959	1	0.255837	-0.10844	-0.20745	-0.08144	0.107563	0.097792	0.247151	0.274843	0.290788	0.267559	0.31897	-0.0108	0.300864	0.255961	0.292963	-0.14759	-0.16351
rsad2	0.96307	0.618414	-0.18158	0.255837	1	0.284022	-0.6669	-0.38713	0.072798	0.473434	0.870755	0.956829	0.968011	0.723128	0.627112	0.330404	0.947046	0.949283	0.66062	0.208316	-0.67213
ifnc3	0.255269	0.025064	-0.00284	-0.10844	0.284022	1	0.048507	-0.17533	-0.36758	0.026042	0.197443	0.230965	0.266765	0.223745	0.337096	0.476863	0.272927	0.289725	0.373555	-0.15568	-0.36948
tnf	-0.70112	-0.50137	0.253285	-0.20745	-0.6669	0.048507	1	0.639789	-0.28141	-0.43649	-0.6797	-0.66268	-0.62109	-0.43265	-0.40072	0.092146	-0.55599	-0.65495	-0.38827	-0.34503	0.505921
il1b	-0.47598	-0.21989	0.165172	-0.08144	-0.38713	-0.17533	0.639789	1	-0.14118	-0.45742	-0.46295	-0.42072	-0.35133	-0.3177	-0.2844	0.166865	-0.30553	-0.43914	-0.26376	-0.37896	0.374076
irf8	0.068768	0.28344	-0.20286	0.107563	0.072798	-0.36758	-0.28141	-0.14118	1	0.544727	0.014048	0.191474	0.000961	0.351617	-0.00272	-0.42183	-0.00125	0.153777	0.078487	0.573014	0.210063
mhc class II	0.455564	0.510124	-0.11641	0.097792	0.473434	0.026042	-0.43649	-0.45742	0.544727	1	0.404001	0.522963	0.367245	0.621605	0.187168	-0.15384	0.356843	0.505354	0.327765	0.69706	-0.16137
gzma	0.843668	0.584331	-0.10303	0.247151	0.870755	0.197443	-0.6797	-0.46295	0.014048	0.404001	1	0.859758	0.846178	0.464748	0.595851	0.253698	0.791931	0.8547	0.609447	0.180572	-0.74601
rigi	0.946853	0.678008	-0.29327	0.274843	0.956829	0.230965	-0.66268	-0.42072	0.191474	0.522963	0.859758	1	0.927638	0.73627	0.577998	0.26597	0.914496	0.955192	0.635616	0.264974	-0.65702
isg15	0.947097	0.521577	-0.0824	0.290788	0.968011	0.266765	-0.62109	-0.35133	0.000961	0.367245	0.846178	0.927638	1	0.674034	0.697489	0.290675	0.966517	0.893984	0.722135	0.093238	-0.70184
tlr3	0.693558	0.481249	-0.07423	0.267559	0.723128	0.223745	-0.43265	-0.3177	0.351617	0.621605	0.464748	0.73627	0.674034	1	0.483844	0.060427	0.733673	0.726825	0.521969	0.386795	-0.19756
cxcl10	0.636009	0.073356	0.226955	0.31897	0.627112	0.337096	-0.40072	-0.2844	-0.00272	0.187168	0.595851	0.577998	0.697489	0.483844	1	0.028135	0.647906	0.611233	0.866221	-0.06624	-0.64756
saa	0.266564	0.253422	-0.24585	-0.0108	0.330404	0.476863	0.092146	0.166865	-0.42183	-0.15384	0.253698	0.26597	0.290675	0.060427	0.028135	1	0.263019	0.23062	0.066499	-0.39531	-0.18147
ifit5	0.934003	0.511824	-0.07371	0.300864	0.947046	0.272927	-0.55599	-0.30553	-0.00125	0.356843	0.791931	0.914496	0.966517	0.733673	0.647906	0.263019	1	0.889295	0.688336	0.120025	-0.60804
ifi44	0.931619	0.62153	-0.2532	0.255961	0.949283	0.289725	-0.65495	-0.43914	0.153777	0.505354	0.8547	0.955192	0.893984	0.726825	0.611233	0.23062	0.889295	1	0.691769	0.301832	-0.66976
irf1	0.676523	0.221622	0.1386	0.292963	0.66062	0.373555	-0.38827	-0.26376	0.078487	0.327765	0.609447	0.635616	0.722135	0.521969	0.866221	0.066499	0.688336	0.691769	1	0.138496	-0.65841
csf1r	0.209491	0.529806	-0.07668	-0.14759	0.208316	-0.15568	-0.34503	-0.37896	0.573014	0.69706	0.180572	0.264974	0.093238	0.386795	-0.06624	-0.39531	0.120025	0.301832	0.138496	1	-0.0464
**PRV3 dCt**	-0.68181	-0.24201	0.044936	-0.16351	-0.67213	-0.36948	0.505921	0.374076	0.210063	-0.16137	-0.74601	-0.65702	-0.70184	-0.19756	-0.64756	-0.18147	-0.60804	-0.66976	-0.65841	-0.0464	1

Table S6. P-value of Pearson's correlation analysis of 5°C PRV-3 exposed shedders (based on all time points).

	mx	*cd4*	*cd8*	*ifng*	*rsad2*	*ifnc3*	*tnf*	*il1b*	*irf8*	*mhc class II*	*gzma*	*rigi*	*isg15*	*tlr3*	*cxcl10*	*saa*	*ifit5*	*ifi44*	*irf1*	*csf1r*	**PRV3 dCt**
mx	0	0.000478	0.268595	0.147635	0	0.138875	2.72E-06	0.00385	0.694668	0.005958	0	0	0	3.85E-06	4.02E-05	0.121646	0	0	8.14E-06	0.22712	6.49E-06
cd4	0.000478	0	0.039266	0.691652	7.52E-05	0.886356	0.002154	0.204361	0.098945	0.001744	0.000228	7.64E-06	0.001313	0.003425	0.675374	0.141856	0.001673	6.75E-05	0.200722	0.001064	0.161311
cd8	0.268595	0.039266	0	0.525712	0.296515	0.98706	0.142078	0.343018	0.242511	0.505455	0.555867	0.087304	0.637933	0.671732	0.189822	0.154556	0.673878	0.142219	0.427177	0.661531	0.797702
ifng	0.147635	0.691652	0.525712	0	0.137969	0.535213	0.231775	0.641865	0.538534	0.576248	0.152321	0.110069	0.090134	0.120209	0.061812	0.950902	0.079047	0.137772	0.087646	0.397491	0.347974
rsad2	0	7.52E-05	0.296515	0.137969	0	0.098224	1.22E-05	0.021588	0.677711	0.004072	0	0	0	9.3E-07	5.55E-05	0.052558	0	0	1.57E-05	0.229795	9.79E-06
ifnc3	0.138875	0.886356	0.98706	0.535213	0.098224	0	0.782001	0.313729	0.029828	0.88195	0.255579	0.181905	0.121355	0.196331	0.047676	0.003776	0.112672	0.09137	0.027075	0.371853	0.028928
tnf	2.72E-06	0.002154	0.142078	0.231775	1.22E-05	0.782001	0	0.000035	0.10149	0.008755	7.11E-06	1.44E-05	6.85E-05	0.009437	0.017055	0.598565	0.000525	1.96E-05	0.021174	0.042369	0.001931
il1b	0.00385	0.204361	0.343018	0.641865	0.021588	0.313729	0.000035	0	0.418542	0.005732	0.005103	0.011847	0.038497	0.062918	0.097759	0.338026	0.074281	0.00831	0.12577	0.024769	0.026845
irf8	0.694668	0.098945	0.242511	0.538534	0.677711	0.029828	0.10149	0.418542	0	0.000716	0.936161	0.270519	0.995628	0.03833	0.987627	0.011604	0.994334	0.377787	0.654035	0.000321	0.225824
mhc class II	0.005958	0.001744	0.505455	0.576248	0.004072	0.88195	0.008755	0.005732	0.000716	0	0.016088	0.001267	0.02999	6.73E-05	0.281643	0.377603	0.035354	0.001958	0.054589	3.28E-06	0.354409
gzma	0	0.000228	0.555867	0.152321	0	0.255579	7.11E-06	0.005103	0.936161	0.016088	0	0	0	0.004911	0.000159	0.141407	1E-08	0	0.000102	0.299248	2.7E-07
rigi	0	7.64E-06	0.087304	0.110069	0	0.181905	1.44E-05	0.011847	0.270519	0.001267	0	0	0	4.6E-07	0.000277	0.12251	0	0	4.08E-05	0.12397	1.81E-05
isg15	0	0.001313	0.637933	0.090134	0	0.121355	6.85E-05	0.038497	0.995628	0.02999	0	0	0	9.04E-06	3.22E-06	0.090265	0	0	9.7E-07	0.594219	2.63E-06
tlr3	3.85E-06	0.003425	0.671732	0.120209	9.3E-07	0.196331	0.009437	0.062918	0.03833	6.73E-05	0.004911	4.6E-07	9.04E-06	0	0.003231	0.730228	5.3E-07	7.6E-07	0.0013	0.021712	0.255294
cxcl10	4.02E-05	0.675374	0.189822	0.061812	5.55E-05	0.047676	0.017055	0.097759	0.987627	0.281643	0.000159	0.000277	3.22E-06	0.003231	0	0.872539	2.58E-05	9.6E-05	0	0.705401	2.61E-05
saa	0.121646	0.141856	0.154556	0.950902	0.052558	0.003776	0.598565	0.338026	0.011604	0.377603	0.141407	0.12251	0.090265	0.730228	0.872539	0	0.126872	0.182575	0.704283	0.018753	0.296819
ifit5	0	0.001673	0.673878	0.079047	0	0.112672	0.000525	0.074281	0.994334	0.035354	1E-08	0	0	5.3E-07	2.58E-05	0.126872	0	0	4.87E-06	0.492223	0.000107
ifi44	0	6.75E-05	0.142219	0.137772	0	0.09137	1.96E-05	0.00831	0.377787	0.001958	0	0	0	7.6E-07	9.6E-05	0.182575	0	0	4.18E-06	0.078041	1.08E-05
irf1	8.14E-06	0.200722	0.427177	0.087646	1.57E-05	0.027075	0.021174	0.12577	0.654035	0.054589	0.000102	4.08E-05	9.7E-07	0.0013	0	0.704283	4.87E-06	4.18E-06	0	0.427526	1.71E-05
csf1r	0.22712	0.001064	0.661531	0.397491	0.229795	0.371853	0.042369	0.024769	0.000321	3.28E-06	0.299248	0.12397	0.594219	0.021712	0.705401	0.018753	0.492223	0.078041	0.427526	0	0.791259
**PRV3 dCt**	6.49E-06	0.161311	0.797702	0.347974	9.79E-06	0.028928	0.001931	0.026845	0.225824	0.354409	2.7E-07	1.81E-05	2.63E-06	0.255294	2.61E-05	0.296819	0.000107	1.08E-05	1.71E-05	0.791259	0

Table S7. Pearson's correlation analysis of 12°C PRV-3 exposed cohabitants (based on all time points). *As dCt is calculated as Ct[PRV-3]-Ct[Reference gene], the lowest dCt value corresponds to the highest virus load. Therefore, a negative value in the correlation analysis for PRV-3 dCt is a positive correlation.

	mx	*cd4*	*cd8*	*ifng*	*rsad2*	*ifnc3*	*tnf*	*il1b*	*irf8*	*mhc class II*	*gzma*	*rigi*	*isg15*	*tlr3*	*cxcl10*	*saa*	*ifit5*	*ifi44*	*irf1*	*csf1r*	**PRV3 dCt**
mx	1	0.722628	-0.03746	0.391922	0.97788	0.330512	-0.28575	-0.24092	-0.08131	0.541317	0.87766	0.957267	0.912202	0.700276	0.716373	0.263864	0.882426	0.89921	0.816377	0.000346	-0.85419
cd4	0.722628	1	0.072731	0.314971	0.741759	0.265424	-0.26661	-0.22485	-0.18885	0.397879	0.670831	0.678759	0.717135	0.567355	0.635775	0.263713	0.739166	0.600987	0.694619	0.038798	-0.6681
cd8	-0.03746	0.072731	1	0.164788	-0.02799	-0.16856	0.401694	0.587842	-0.76717	0.012919	-0.11944	-0.22711	0.255061	-0.09348	0.349789	-0.075	0.296583	-0.1679	0.353579	-0.52925	0.107923
ifng	0.391922	0.314971	0.164788	1	0.480231	0.506175	-0.12487	-0.12143	-0.28615	-0.21265	0.377886	0.357928	0.564384	0.247246	0.506672	0.467512	0.558358	0.321991	0.561169	-0.4513	-0.48445
rsad2	0.97788	0.741759	-0.02799	0.480231	1	0.438362	-0.32456	-0.27272	-0.13444	0.457558	0.879005	0.940692	0.938582	0.695541	0.761674	0.328881	0.922992	0.864596	0.829445	-0.1043	-0.87753
ifnc3	0.330512	0.265424	-0.16856	0.506175	0.438362	1	-0.17785	-0.23464	-0.11052	-0.19916	0.370666	0.34756	0.376784	0.200251	0.44365	0.38962	0.375541	0.206511	0.312753	-0.39516	-0.5075
tnf	-0.28575	-0.26661	0.401694	-0.12487	-0.32456	-0.17785	1	0.258104	-0.25448	-0.12941	-0.47626	-0.29284	-0.15153	-0.07189	0.054728	0.108262	-0.23679	-0.29199	-0.15843	-0.17087	0.339442
il1b	-0.24092	-0.22485	0.587842	-0.12143	-0.27272	-0.23464	0.258104	1	-0.57166	0.003955	-0.19242	-0.37736	-0.07444	-0.28275	-0.095	0.1149	-0.04337	-0.11044	0.169334	-0.276	0.447177
irf8	-0.08131	-0.18885	-0.76717	-0.28615	-0.13444	-0.11052	-0.25448	-0.57166	1	0.207109	-0.03981	0.127137	-0.40298	0.110762	-0.57613	-0.26146	-0.44026	0.085335	-0.49788	0.804457	-0.00539
mhc class II	0.541317	0.397879	0.012919	-0.21265	0.457558	-0.19916	-0.12941	0.003955	0.207109	1	0.510807	0.517175	0.362823	0.506271	0.139112	-0.2503	0.322448	0.567674	0.369892	0.531237	-0.39392
gzma	0.87766	0.670831	-0.11944	0.377886	0.879005	0.370666	-0.47626	-0.19242	-0.03981	0.510807	1	0.860849	0.812583	0.542018	0.562156	0.284199	0.791449	0.845161	0.772302	-0.02203	-0.81268
rigi	0.957267	0.678759	-0.22711	0.357928	0.940692	0.34756	-0.29284	-0.37736	0.127137	0.517175	0.860849	1	0.814214	0.754973	0.617667	0.303868	0.77658	0.911152	0.680181	0.149025	-0.80084
isg15	0.912202	0.717135	0.255061	0.564384	0.938582	0.376784	-0.15153	-0.07444	-0.40298	0.362823	0.812583	0.814214	1	0.614491	0.833589	0.351769	0.984226	0.774999	0.940418	-0.34317	-0.81883
tlr3	0.700276	0.567355	-0.09348	0.247246	0.695541	0.200251	-0.07189	-0.28275	0.110762	0.506271	0.542018	0.754973	0.614491	1	0.491951	0.307868	0.601165	0.664078	0.505018	0.233194	-0.55312
cxcl10	0.716373	0.635775	0.349789	0.506672	0.761674	0.44365	0.054728	-0.095	-0.57613	0.139112	0.562156	0.617667	0.833589	0.491951	1	0.315261	0.832725	0.473699	0.738383	-0.47378	-0.5918
saa	0.263864	0.263713	-0.075	0.467512	0.328881	0.38962	0.108262	0.1149	-0.26146	-0.2503	0.284199	0.303868	0.351769	0.307868	0.315261	1	0.327902	0.36093	0.365222	-0.28927	-0.22223
ifit5	0.882426	0.739166	0.296583	0.558358	0.922992	0.375541	-0.23679	-0.04337	-0.44026	0.322448	0.791449	0.77658	0.984226	0.601165	0.832725	0.327902	1	0.733578	0.928888	-0.35945	-0.78457
ifi44	0.89921	0.600987	-0.1679	0.321991	0.864596	0.206511	-0.29199	-0.11044	0.085335	0.567674	0.845161	0.911152	0.774999	0.664078	0.473699	0.36093	0.733578	1	0.722144	0.182658	-0.71904
irf1	0.816377	0.694619	0.353579	0.561169	0.829445	0.312753	-0.15843	0.169334	-0.49788	0.369892	0.772302	0.680181	0.940418	0.505018	0.738383	0.365222	0.928888	0.722144	1	-0.37307	-0.72605
csf1r	0.000346	0.038798	-0.52925	-0.4513	-0.1043	-0.39516	-0.17087	-0.276	0.804457	0.531237	-0.02203	0.149025	-0.34317	0.233194	-0.47378	-0.28927	-0.35945	0.182658	-0.37307	1	0.103202
**PRV3 dCt**	-0.85419	-0.6681	0.107923	-0.48445	-0.87753	-0.5075	0.339442	0.447177	-0.00539	-0.39392	-0.81268	-0.80084	-0.81883	-0.55312	-0.5918	-0.22223	-0.78457	-0.71904	-0.72605	0.103202	1

Table S8. P-value of Pearson's correlation analysis of 12°C PRV-3 exposed cohabitants (based on all time points).

	mx	*cd4*	*cd8*	*ifng*	*rsad2*	*ifnc3*	*tnf*	*il1b*	*irf8*	*mhc class II*	*gzma*	*rigi*	*isg15*	*tlr3*	*cxcl10*	*saa*	*ifit5*	*ifi44*	*irf1*	*csf1r*	**PRV3 dCt**
mx	0	0.000216	0.871931	0.078898	0	0.143372	0.209237	0.292785	0.726051	0.011269	1.7E-07	0	1E-08	0.000408	0.000259	0.247778	1.2E-07	3E-08	6.38E-06	0.998813	8.3E-07
cd4	0.000216	0	0.754051	0.164319	0.000119	0.244889	0.242717	0.327115	0.412306	0.074058	0.000873	0.000717	0.000254	0.00731	0.001951	0.248058	0.000129	0.00396	0.000476	0.867395	0.000933
cd8	0.871931	0.754051	0	0.475342	0.904155	0.465157	0.071078	0.005071	4.95E-05	0.955676	0.606074	0.322157	0.264488	0.686921	0.1201	0.746631	0.191723	0.466933	0.115864	0.01362	0.641468
ifng	0.078898	0.164319	0.475342	0	0.027569	0.019217	0.589677	0.600049	0.208576	0.354715	0.09123	0.111138	0.007693	0.279906	0.01908	0.032598	0.008522	0.15461	0.008127	0.040021	0.026044
rsad2	0	0.000119	0.904155	0.027569	0	0.046841	0.15116	0.231669	0.561225	0.037014	1.6E-07	0	0	0.000464	6.03E-05	0.145477	0	4.3E-07	3.34E-06	0.652754	1.7E-07
ifnc3	0.143372	0.244889	0.465157	0.019217	0.046841	0	0.440535	0.305926	0.633429	0.386769	0.098102	0.122642	0.092255	0.384116	0.043953	0.08083	0.093422	0.369112	0.167471	0.076241	0.018852
tnf	0.209237	0.242717	0.071078	0.589677	0.15116	0.440535	0	0.258633	0.26562	0.576104	0.02907	0.19766	0.512026	0.756811	0.813733	0.640417	0.301385	0.199018	0.492757	0.458977	0.132227
il1b	0.292785	0.327115	0.005071	0.600049	0.231669	0.305926	0.258633	0	0.006782	0.986426	0.403365	0.091717	0.74846	0.214268	0.682094	0.619934	0.851938	0.633677	0.463074	0.225872	0.042105
irf8	0.726051	0.412306	4.95E-05	0.208576	0.561225	0.633429	0.26562	0.006782	0	0.367696	0.863972	0.582885	0.070092	0.632673	0.006268	0.252264	0.04579	0.713036	0.021629	1.1E-05	0.981505
mhc class II	0.011269	0.074058	0.955676	0.354715	0.037014	0.386769	0.576104	0.986426	0.367696	0	0.017967	0.016355	0.105988	0.01919	0.54758	0.273814	0.153993	0.00727	0.09886	0.013208	0.077247
gzma	1.7E-07	0.000873	0.606074	0.09123	1.6E-07	0.098102	0.02907	0.403365	0.863972	0.017967	0	5.5E-07	7.63E-06	0.011144	0.007992	0.211827	1.93E-05	1.42E-06	4.09E-05	0.924493	7.59E-06
rigi	0	0.000717	0.322157	0.111138	0	0.122642	0.19766	0.091717	0.582885	0.016355	5.5E-07	0	7.07E-06	7.63E-05	0.002849	0.18052	3.48E-05	1E-08	0.000692	0.519111	1.29E-05
isg15	1E-08	0.000254	0.264488	0.007693	0	0.092255	0.512026	0.74846	0.070092	0.105988	7.63E-06	7.07E-06	0	0.003038	2.69E-06	0.117873	0	3.7E-05	0	0.127765	5.67E-06
tlr3	0.000408	0.00731	0.686921	0.279906	0.000464	0.384116	0.756811	0.214268	0.632673	0.01919	0.011144	7.63E-05	0.003038	0	0.023498	0.174562	0.003947	0.001027	0.01954	0.309006	0.009301
cxcl10	0.000259	0.001951	0.1201	0.01908	6.03E-05	0.043953	0.813733	0.682094	0.006268	0.54758	0.007992	0.002849	2.69E-06	0.023498	0	0.163909	2.82E-06	0.030069	0.000132	0.030038	0.004712
saa	0.247778	0.248058	0.746631	0.032598	0.145477	0.08083	0.640417	0.619934	0.252264	0.273814	0.211827	0.18052	0.117873	0.174562	0.163909	0	0.146752	0.107958	0.103529	0.203437	0.332942
ifit5	1.2E-07	0.000129	0.191723	0.008522	0	0.093422	0.301385	0.851938	0.04579	0.153993	1.93E-05	3.48E-05	0	0.003947	2.82E-06	0.146752	0	0.000154	0	0.109513	2.55E-05
ifi44	3E-08	0.00396	0.466933	0.15461	4.3E-07	0.369112	0.199018	0.633677	0.713036	0.00727	1.42E-06	1E-08	3.7E-05	0.001027	0.030069	0.107958	0.000154	0	0.000219	0.428074	0.00024
irf1	6.38E-06	0.000476	0.115864	0.008127	3.34E-06	0.167471	0.492757	0.463074	0.021629	0.09886	4.09E-05	0.000692	0	0.01954	0.000132	0.103529	0	0.000219	0	0.095775	0.000194
csf1r	0.998813	0.867395	0.01362	0.040021	0.652754	0.076241	0.458977	0.225872	1.1E-05	0.013208	0.924493	0.519111	0.127765	0.309006	0.030038	0.203437	0.109513	0.428074	0.095775	0	0.656203
**PRV3 dCt**	8.3E-07	0.000933	0.641468	0.026044	1.7E-07	0.018852	0.132227	0.042105	0.981505	0.077247	7.59E-06	1.29E-05	5.67E-06	0.009301	0.004712	0.332942	2.55E-05	0.00024	0.000194	0.656203	0

Table S9. Pearson's correlation analysis of 12°C PRV-3 exposed shedders (based on all time points). *As dCt is calculated as Ct[PRV-3]-Ct[Reference gene], the lowest dCt value corresponds to the highest virus load. Therefore, a negative value in the correlation analysis for PRV-3 dCt is a positive correlation.

	mx	*cd4*	*cd8*	*ifng*	*rsad2*	*ifnc3*	*tnf*	*il1b*	*irf8*	*mhc class II*	*gzma*	*rigi*	*isg15*	*tlr3*	*cxcl10*	*saa*	*ifit5*	*ifi44*	*irf1*	*csf1r*	**PRV3 dCt**
mx	1	-0.10583	-0.37156	0.275527	0.875626	0.440674	-0.10631	-0.13631	0.23417	-0.149	-0.17301	0.909506	0.772776	0.671051	0.718273	0.346054	0.748587	0.888428	0.541349	-0.10579	-0.51135
cd4	-0.10583	1	0.298985	-0.17946	-0.29979	0.205619	0.361091	0.0044	0.631095	0.373581	0.157772	-0.03819	-0.34266	-0.16347	-0.25851	-0.23256	-0.37246	-0.1885	0.045348	0.490275	0.102352
cd8	-0.37156	0.298985	1	-0.31842	-0.54093	-0.2067	0.258226	-0.14385	0.455892	0.578957	-0.16921	-0.27355	-0.67192	-0.11841	-0.35187	-0.5406	-0.39609	-0.50494	-0.52023	0.616784	0.595373
ifng	0.275527	-0.17946	-0.31842	1	0.253806	0.231683	-0.22318	0.174734	-0.05868	-0.50617	0.367855	0.104415	0.39481	-0.13245	0.25508	-0.07988	0.300595	0.175549	0.449568	-0.39566	-0.42468
rsad2	0.875626	-0.29979	-0.54093	0.253806	1	0.507873	-0.07603	-0.05536	-0.0106	-0.12875	-0.29551	0.84603	0.929875	0.740638	0.813944	0.539469	0.890068	0.834731	0.725736	-0.44157	-0.57978
ifnc3	0.440674	0.205619	-0.2067	0.231683	0.507873	1	0.488899	0.294213	0.052175	-0.06368	-0.08962	0.350517	0.457183	0.32061	0.440654	0.552275	0.445479	0.28676	0.584292	-0.43653	-0.46644
tnf	-0.10631	0.361091	0.258226	-0.22318	-0.07603	0.488899	1	0.373791	0.259409	0.294193	-0.39045	-0.07785	-0.20143	0.22013	0.052793	0.029283	-0.0133	-0.12837	0.254692	0.066577	0.254933
il1b	-0.13631	0.0044	-0.14385	0.174734	-0.05536	0.294213	0.373791	1	0.060118	-0.36421	-0.28813	-0.14337	-0.00427	0.035647	0.324774	0.231394	0.048509	-0.02768	0.181999	-0.38476	-0.00701
irf8	0.23417	0.631095	0.455892	-0.05868	-0.0106	0.052175	0.259409	0.060118	1	0.488081	-0.25115	0.307452	-0.12511	0.226996	0.175673	-0.38492	0.042065	0.02877	0.119543	0.573202	0.255949
mhc class II	-0.149	0.373581	0.578957	-0.50617	-0.12875	-0.06368	0.294193	-0.36421	0.488081	1	-0.31234	0.06834	-0.36558	0.172953	-0.20826	-0.29853	-0.18944	-0.24777	-0.09628	0.49558	0.493636
gzma	-0.17301	0.157772	-0.16921	0.367855	-0.29551	-0.08962	-0.39045	-0.28813	-0.25115	-0.31234	1	-0.44843	-0.14669	-0.7822	-0.57299	0.086467	-0.45815	-0.28134	-0.25052	-0.09882	-0.42178
rigi	0.909506	-0.03819	-0.27355	0.104415	0.84603	0.350517	-0.07785	-0.14337	0.307452	0.06834	-0.44843	1	0.690755	0.844533	0.790746	0.188385	0.736717	0.86342	0.543574	0.039545	-0.28409
isg15	0.772776	-0.34266	-0.67192	0.39481	0.929875	0.457183	-0.20143	-0.00427	-0.12511	-0.36558	-0.14669	0.690755	1	0.557027	0.748241	0.524652	0.899329	0.751721	0.753482	-0.59743	-0.69126
tlr3	0.671051	-0.16347	-0.11841	-0.13245	0.740638	0.32061	0.22013	0.035647	0.226996	0.172953	-0.7822	0.844533	0.557027	1	0.825506	0.198427	0.751105	0.685681	0.512623	-0.00039	0.011367
cxcl10	0.718273	-0.25851	-0.35187	0.25508	0.813944	0.440654	0.052793	0.324774	0.175673	-0.20826	-0.57299	0.790746	0.748241	0.825506	1	0.355409	0.830935	0.673802	0.608412	-0.30972	-0.31383
saa	0.346054	-0.23256	-0.5406	-0.07988	0.539469	0.552275	0.029283	0.231394	-0.38492	-0.29853	0.086467	0.188385	0.524652	0.198427	0.355409	1	0.331839	0.328458	0.26418	-0.65613	-0.66672
ifit5	0.748587	-0.37246	-0.39609	0.300595	0.890068	0.445479	-0.0133	0.048509	0.042065	-0.18944	-0.45815	0.736717	0.899329	0.751105	0.830935	0.331839	1	0.722336	0.724337	-0.47098	-0.36228
ifi44	0.888428	-0.1885	-0.50494	0.175549	0.834731	0.28676	-0.12837	-0.02768	0.02877	-0.24777	-0.28134	0.86342	0.751721	0.685681	0.673802	0.328458	0.722336	1	0.541537	-0.19992	-0.43172
irf1	0.541349	0.045348	-0.52023	0.449568	0.725736	0.584292	0.254692	0.181999	0.119543	-0.09628	-0.25052	0.543574	0.753482	0.512623	0.608412	0.26418	0.724337	0.541537	1	-0.45213	-0.44852
csf1r	-0.10579	0.490275	0.616784	-0.39566	-0.44157	-0.43653	0.066577	-0.38476	0.573202	0.49558	-0.09882	0.039545	-0.59743	-0.00039	-0.30972	-0.65613	-0.47098	-0.19992	-0.45213	1	0.555591
**PRV3 dCt**	-0.51135	0.102352	0.595373	-0.42468	-0.57978	-0.46644	0.254933	-0.00701	0.255949	0.493636	-0.42178	-0.28409	-0.69126	0.011367	-0.31383	-0.66672	-0.36228	-0.43172	-0.44852	0.555591	1

Table S10. P-value of Pearson's correlation analysis of 12°C PRV-3 exposed shedders (based on all time points).

	mx	*cd4*	*cd8*	*ifng*	*rsad2*	*ifnc3*	*tnf*	*il1b*	*irf8*	*mhc class II*	*gzma*	*rigi*	*isg15*	*tlr3*	*cxcl10*	*saa*	*ifit5*	*ifi44*	*irf1*	*csf1r*	**PRV3 dCt**
mx	0	0.69647	0.156478	0.301663	8.83E-06	0.087556	0.69517	0.614709	0.382703	0.581797	0.521667	1.05E-06	0.000449	0.00443	0.001724	0.189202	0.000849	4.28E-06	0.030337	0.696586	0.042921
cd4	0.69647	0	0.260631	0.506012	0.259283	0.444881	0.169411	0.987098	0.008751	0.154061	0.559509	0.888339	0.193866	0.545228	0.333674	0.386066	0.1554	0.484471	0.867557	0.053857	0.706033
cd8	0.156478	0.260631	0	0.229383	0.030492	0.442449	0.33423	0.595065	0.075938	0.018777	0.531001	0.305288	0.004359	0.662279	0.18137	0.030612	0.128825	0.046053	0.038847	0.01093	0.014964
ifng	0.301663	0.506012	0.229383	0	0.342859	0.38792	0.406056	0.517472	0.829082	0.045437	0.160978	0.70036	0.130177	0.624859	0.340358	0.768716	0.257948	0.515487	0.080622	0.129281	0.10108
rsad2	8.83E-06	0.259283	0.030492	0.342859	0	0.044599	0.779591	0.838627	0.968912	0.634631	0.266483	3.62E-05	1.9E-07	0.001032	0.000125	0.031031	3.87E-06	5.76E-05	0.00146	0.086841	0.01857
ifnc3	0.087556	0.444881	0.442449	0.38792	0.044599	0	0.054636	0.268684	0.847822	0.81476	0.741345	0.183178	0.075007	0.226012	0.087573	0.026534	0.083759	0.281562	0.017464	0.090933	0.068567
tnf	0.69517	0.169411	0.33423	0.406056	0.779591	0.054636	0	0.153811	0.331941	0.268718	0.134856	0.774426	0.454414	0.412664	0.846037	0.914273	0.961008	0.635635	0.341119	0.806474	0.340648
il1b	0.614709	0.987098	0.595065	0.517472	0.838627	0.268684	0.153811	0	0.824964	0.165481	0.279161	0.596307	0.987469	0.895726	0.219699	0.38853	0.858409	0.918944	0.499924	0.141145	0.979436
irf8	0.382703	0.008751	0.075938	0.829082	0.968912	0.847822	0.331941	0.824964	0	0.055103	0.348105	0.246709	0.644329	0.39786	0.515187	0.140962	0.877074	0.915769	0.659233	0.020279	0.33866
mhc class II	0.581797	0.154061	0.018777	0.045437	0.634631	0.81476	0.268718	0.165481	0.055103	0	0.238888	0.801444	0.163778	0.521814	0.438927	0.261389	0.48225	0.354851	0.722797	0.050928	0.051987
gzma	0.521667	0.559509	0.531001	0.160978	0.266483	0.741345	0.134856	0.279161	0.348105	0.238888	0	0.081483	0.587738	0.000343	0.020335	0.750174	0.074316	0.291159	0.349365	0.715778	0.103688
rigi	1.05E-06	0.888339	0.305288	0.70036	3.62E-05	0.183178	0.774426	0.596307	0.246709	0.801444	0.081483	0	0.00305	3.86E-05	0.000265	0.484734	0.001133	1.64E-05	0.029531	0.884391	0.28626
isg15	0.000449	0.193866	0.004359	0.130177	1.9E-07	0.075007	0.454414	0.987469	0.644329	0.163778	0.587738	0.00305	0	0.024999	0.000857	0.036933	2.15E-06	0.000785	0.000751	0.014532	0.00302
tlr3	0.00443	0.545228	0.662279	0.624859	0.001032	0.226012	0.412664	0.895726	0.39786	0.521814	0.000343	3.86E-05	0.024999	0	8.22E-05	0.461306	0.000797	0.003366	0.042318	0.998866	0.966672
cxcl10	0.001724	0.333674	0.18137	0.340358	0.000125	0.087573	0.846037	0.219699	0.515187	0.438927	0.020335	0.000265	0.000857	8.22E-05	0	0.17672	6.69E-05	0.004211	0.01239	0.243056	0.236533
saa	0.189202	0.386066	0.030612	0.768716	0.031031	0.026534	0.914273	0.38853	0.140962	0.261389	0.750174	0.484734	0.036933	0.461306	0.17672	0	0.209248	0.214208	0.322803	0.005776	0.004791
ifit5	0.000849	0.1554	0.128825	0.257948	3.87E-06	0.083759	0.961008	0.858409	0.877074	0.48225	0.074316	0.001133	2.15E-06	0.000797	6.69E-05	0.209248	0	0.001576	0.001507	0.065564	0.167913
ifi44	4.28E-06	0.484471	0.046053	0.515487	5.76E-05	0.281562	0.635635	0.918944	0.915769	0.354851	0.291159	1.64E-05	0.000785	0.003366	0.004211	0.214208	0.001576	0	0.030268	0.457864	0.094956
irf1	0.030337	0.867557	0.038847	0.080622	0.00146	0.017464	0.341119	0.499924	0.659233	0.722797	0.349365	0.029531	0.000751	0.042318	0.01239	0.322803	0.001507	0.030268	0	0.078698	0.081416
csf1r	0.696586	0.053857	0.01093	0.129281	0.086841	0.090933	0.806474	0.141145	0.020279	0.050928	0.715778	0.884391	0.014532	0.998866	0.243056	0.005776	0.065564	0.457864	0.078698	0	0.025455
**PRV3 dCt**	0.042921	0.706033	0.014964	0.10108	0.01857	0.068567	0.340648	0.979436	0.33866	0.051987	0.103688	0.28626	0.00302	0.966672	0.236533	0.004791	0.167913	0.094956	0.081416	0.025455	0

Table S11. Pearson's correlation analysis of 18°C PRV-3 exposed cohabitants (based on all time points). *As dCt is calculated as Ct[PRV-3]-Ct[Reference gene], the lowest dCt value corresponds to the highest virus load. Therefore, a negative value in the correlation analysis for PRV-3 dCt is a positive correlation.

	mx	*cd4*	*cd8*	*ifng*	*rsad2*	*ifnc3*	*tnf*	*il1b*	*irf8*	*mhc class II*	*gzma*	*rigi*	*isg15*	*tlr3*	*cxcl10*	*saa*	*ifit5*	*ifi44*	*irf1*	*csf1r*	**PRV3 dCt**
mx	1	0.437447	0.031962	0.41694	0.818172	0.321793	-0.25605	-0.24223	0.110478	0.226496	0.497689	0.896483	0.735466	0.565038	0.788517	0.017344	0.730391	0.698841	0.601284	0.124976	-0.40769
cd4	0.437447	1	0.477072	0.522541	0.499852	-0.04696	0.020221	0.146998	-0.29447	0.568799	0.440997	0.386909	0.539636	0.403419	0.355944	0.136192	0.634664	0.33602	0.623333	0.258209	-0.28668
cd8	0.031962	0.477072	1	0.467745	0.246471	-0.35277	0.177543	0.458562	-0.45871	0.817506	0.325457	0.016836	0.356835	0.385628	0.101026	-0.30822	0.479688	0.090617	0.620121	-0.14188	-0.11777
ifng	0.41694	0.522541	0.467745	1	0.613193	-0.08646	-0.33351	0.386026	-0.40848	0.440523	0.634168	0.236969	0.599886	0.284847	0.386345	0.02496	0.622511	0.160774	0.638328	-0.31209	-0.51142
rsad2	0.818172	0.499852	0.246471	0.613193	1	0.260697	-0.31553	0.030332	-0.21229	0.388545	0.760834	0.767624	0.960865	0.499222	0.839865	0.1176	0.897911	0.62869	0.835774	-0.19453	-0.58274
ifnc3	0.321793	-0.04696	-0.35277	-0.08646	0.260697	1	-0.25079	-0.23055	0.162805	-0.13537	0.126922	0.367284	0.328202	0.307878	0.286818	0.226606	0.241415	0.178375	0.095241	-0.15495	-0.09624
tnf	-0.25605	0.020221	0.177543	-0.33351	-0.31553	-0.25079	1	0.212836	0.216776	0.12687	-0.39228	-0.08632	-0.30256	0.104089	-0.14849	-0.0542	-0.22199	0.094339	-0.18995	0.410818	0.255648
il1b	-0.24223	0.146998	0.458562	0.386026	0.030332	-0.23055	0.212836	1	-0.58796	0.194466	0.180591	-0.35337	0.082135	-0.1639	-0.073	-0.18996	0.115531	-0.02998	0.251723	-0.28367	-0.13262
irf8	0.110478	-0.29447	-0.45871	-0.40848	-0.21229	0.162805	0.216776	-0.58796	1	-0.20371	-0.45022	0.305908	-0.33207	0.308548	-0.06732	-0.1722	-0.37655	-0.11471	-0.44766	0.425863	0.181479
mhc class II	0.226496	0.568799	0.817506	0.440523	0.388545	-0.13537	0.12687	0.194466	-0.20371	1	0.423778	0.31842	0.473981	0.6304	0.213018	-0.30394	0.551862	0.280796	0.654451	0.132475	-0.18211
gzma	0.497689	0.440997	0.325457	0.634168	0.760834	0.126922	-0.39228	0.180591	-0.45022	0.423778	1	0.436656	0.773052	0.102564	0.632646	0.271754	0.746825	0.453044	0.801931	-0.32404	-0.5101
rigi	0.896483	0.386909	0.016836	0.236969	0.767624	0.367284	-0.08632	-0.35337	0.305908	0.31842	0.436656	1	0.696112	0.660174	0.775686	-0.07	0.672448	0.695867	0.531832	0.237948	-0.34796
isg15	0.735466	0.539636	0.356835	0.599886	0.960865	0.328202	-0.30256	0.082135	-0.33207	0.473981	0.773052	0.696112	1	0.514209	0.792324	0.126147	0.960054	0.610146	0.895565	-0.27793	-0.56021
tlr3	0.565038	0.403419	0.385628	0.284847	0.499222	0.307878	0.104089	-0.1639	0.308548	0.6304	0.102564	0.660174	0.514209	1	0.406477	-0.2936	0.529791	0.41147	0.479996	0.220076	-0.14232
cxcl10	0.788517	0.355944	0.101026	0.386345	0.839865	0.286818	-0.14849	-0.073	-0.06732	0.213018	0.632646	0.775686	0.792324	0.406477	1	0.095096	0.761413	0.716589	0.697035	-0.02499	-0.57468
saa	0.017344	0.136192	-0.30822	0.02496	0.1176	0.226606	-0.0542	-0.18996	-0.1722	-0.30394	0.271754	-0.07	0.126147	-0.2936	0.095096	1	0.072181	-0.07661	0.044039	-0.17436	-0.10956
ifit5	0.730391	0.634664	0.479688	0.622511	0.897911	0.241415	-0.22199	0.115531	-0.37655	0.551862	0.746825	0.672448	0.960054	0.529791	0.761413	0.072181	1	0.615166	0.931153	-0.2557	-0.52529
ifi44	0.698841	0.33602	0.090617	0.160774	0.62869	0.178375	0.094339	-0.02998	-0.11471	0.280796	0.453044	0.695867	0.610146	0.41147	0.716589	-0.07661	0.615166	1	0.5664	0.266356	-0.25301
irf1	0.601284	0.623333	0.620121	0.638328	0.835774	0.095241	-0.18995	0.251723	-0.44766	0.654451	0.801931	0.531832	0.895565	0.479996	0.697035	0.044039	0.931153	0.5664	1	-0.26503	-0.45686
csf1r	0.124976	0.258209	-0.14188	-0.31209	-0.19453	-0.15495	0.410818	-0.28367	0.425863	0.132475	-0.32404	0.237948	-0.27793	0.220076	-0.02499	-0.17436	-0.2557	0.266356	-0.26503	1	0.128658
**PRV3 dCt**	-0.40769	-0.28668	-0.11777	-0.51142	-0.58274	-0.09624	0.255648	-0.13262	0.181479	-0.18211	-0.5101	-0.34796	-0.56021	-0.14232	-0.57468	-0.10956	-0.52529	-0.25301	-0.45686	0.128658	1

Table S12. P-value of Pearson's correlation analysis of 18°C PRV-3 exposed cohabitants (based on all time points)

	mx	*cd4*	*cd8*	*ifng*	*rsad2*	*ifnc3*	*tnf*	*il1b*	*irf8*	*mhc class II*	*gzma*	*rigi*	*isg15*	*tlr3*	*cxcl10*	*saa*	*ifit5*	*ifi44*	*irf1*	*csf1r*	**PRV3 dCt**
mx	0	0.036849	0.884889	0.047787	1.85E-06	0.134299	0.238292	0.265457	0.615789	0.29869	0.015669	1E-08	6.37E-05	0.004964	7.84E-06	0.937393	7.59E-05	0.000208	0.002408	0.569917	0.053478
cd4	0.036849	0	0.021344	0.010527	0.015153	0.831494	0.927034	0.503295	0.172591	0.004622	0.035174	0.068174	0.007869	0.056279	0.095523	0.535503	0.001142	0.116973	0.001485	0.234213	0.184765
cd8	0.884889	0.021344	0	0.024405	0.256913	0.098726	0.417679	0.027753	0.027698	1.91E-06	0.129671	0.939223	0.094636	0.069171	0.646477	0.152479	0.020544	0.68093	0.001597	0.518419	0.592513
ifng	0.047787	0.010527	0.024405	0	0.001863	0.694878	0.119902	0.06886	0.052974	0.035394	0.001155	0.276292	0.00248	0.187709	0.068612	0.909994	0.001513	0.463659	0.001047	0.147123	0.012622
rsad2	1.85E-06	0.015153	0.256913	0.001863	0	0.229577	0.142486	0.890726	0.330804	0.066916	0.000025	1.91E-05	0	0.015302	5.4E-07	0.593065	1E-08	0.001313	6.9E-07	0.373761	0.003523
ifnc3	0.134299	0.831494	0.098726	0.694878	0.229577	0	0.248405	0.289895	0.457955	0.537986	0.563875	0.084699	0.12628	0.152952	0.18454	0.298451	0.267114	0.415467	0.665542	0.480222	0.662244
tnf	0.238292	0.927034	0.417679	0.119902	0.142486	0.248405	0	0.329541	0.320455	0.564038	0.06411	0.695327	0.160538	0.636468	0.498909	0.805992	0.308654	0.668534	0.385334	0.051498	0.23905
il1b	0.265457	0.503295	0.027753	0.06886	0.890726	0.289895	0.329541	0	0.003172	0.373922	0.4096	0.098112	0.709471	0.454885	0.740621	0.385309	0.599629	0.892003	0.246588	0.189613	0.546364
irf8	0.615789	0.172591	0.027698	0.052974	0.330804	0.457955	0.320455	0.003172	0	0.351181	0.031104	0.155734	0.121606	0.152014	0.760205	0.432065	0.076562	0.602259	0.032192	0.042753	0.407262
mhc class II	0.29869	0.004622	1.91E-06	0.035394	0.066916	0.537986	0.564038	0.373922	0.351181	0	0.04389	0.138663	0.022322	0.001262	0.329119	0.15855	0.006332	0.194339	0.000704	0.546799	0.405595
gzma	0.015669	0.035174	0.129671	0.001155	0.000025	0.563875	0.06411	0.4096	0.031104	0.04389	0	0.03723	1.53E-05	0.641446	0.001198	0.209706	4.25E-05	0.029936	4.2E-06	0.131451	0.012891
rigi	1E-08	0.068174	0.939223	0.276292	1.91E-05	0.084699	0.695327	0.098112	0.155734	0.138663	0.03723	0	0.000225	0.000608	1.37E-05	0.750949	0.00044	0.000227	0.009004	0.274254	0.103731
isg15	6.37E-05	0.007869	0.094636	0.00248	0	0.12628	0.160538	0.709471	0.121606	0.022322	1.53E-05	0.000225	0	0.012067	6.6E-06	0.566278	0	0.001991	1E-08	0.199119	0.005434
tlr3	0.004964	0.056279	0.069171	0.187709	0.015302	0.152952	0.636468	0.454885	0.152014	0.001262	0.641446	0.000608	0.012067	0	0.054264	0.17392	0.009322	0.051093	0.020451	0.312961	0.517123
cxcl10	7.84E-06	0.095523	0.646477	0.068612	5.4E-07	0.18454	0.498909	0.740621	0.760205	0.329119	0.001198	1.37E-05	6.6E-06	0.054264	0	0.666025	2.44E-05	0.00012	0.000219	0.909901	0.004128
saa	0.937393	0.535503	0.152479	0.909994	0.593065	0.298451	0.805992	0.385309	0.432065	0.15855	0.209706	0.750949	0.566278	0.17392	0.666025	0	0.743448	0.728268	0.841856	0.426205	0.618738
ifit5	7.59E-05	0.001142	0.020544	0.001513	1E-08	0.267114	0.308654	0.599629	0.076562	0.006332	4.25E-05	0.00044	0	0.009322	2.44E-05	0.743448	0	0.001783	0	0.238949	0.010056
ifi44	0.000208	0.116973	0.68093	0.463659	0.001313	0.415467	0.668534	0.892003	0.602259	0.194339	0.029936	0.000227	0.001991	0.051093	0.00012	0.728268	0.001783	0	0.004838	0.219257	0.244105
irf1	0.002408	0.001485	0.001597	0.001047	6.9E-07	0.665542	0.385334	0.246588	0.032192	0.000704	4.2E-06	0.009004	1E-08	0.020451	0.000219	0.841856	0	0.004838	0	0.221656	0.028414
csf1r	0.569917	0.234213	0.518419	0.147123	0.373761	0.480222	0.051498	0.189613	0.042753	0.546799	0.131451	0.274254	0.199119	0.312961	0.909901	0.426205	0.238949	0.219257	0.221656	0	0.558512
**PRV3 dCt**	0.053478	0.184765	0.592513	0.012622	0.003523	0.662244	0.23905	0.546364	0.407262	0.405595	0.012891	0.103731	0.005434	0.517123	0.004128	0.618738	0.010056	0.244105	0.028414	0.558512	0

Table S13. Pearson's correlation analysis of 18°C PRV-3 exposed shedders (based on all time points). *As dCt is calculated as Ct[PRV-3]-Ct[Reference gene], the lowest dCt value corresponds to the highest virus load. Therefore, a negative value in the correlation analysis for PRV-3 dCt is a positive correlation.

	mx	*cd4*	*cd8*	*ifng*	*rsad2*	*ifnc3*	*tnf*	*il1b*	*irf8*	*mhc class II*	*gzma*	*rigi*	*isg15*	*tlr3*	*cxcl10*	*saa*	*ifit5*	*ifi44*	*irf1*	*csf1r*	**PRV3 dCt**
mx	1	-0.01214	-0.35144	0.0682	0.859712	0.071066	0.027486	0.020968	-0.14936	0.050158	0.76408	0.883531	0.770092	0.32294	0.80199	0.091341	0.812858	0.632442	0.019017	-0.26926	-0.58573
cd4	-0.01214	1	-0.37069	-0.05792	0.25798	0.288268	0.645835	0.234914	0.364865	0.544349	-0.37522	0.31021	-0.20241	0.223448	0.0416	0.521815	-0.2759	0.223173	-0.1094	0.558338	0.496761
cd8	-0.35144	-0.37069	1	0.290378	-0.63007	-0.04704	-0.13686	-0.16726	0.346211	-0.06263	-0.11692	-0.4588	-0.5029	0.07937	-0.32851	-0.4387	-0.25564	-0.25918	0.275263	0.350935	0.100495
ifng	0.0682	-0.05792	0.290378	1	-0.02834	-0.09362	-0.04708	-0.32438	0.308945	-0.17581	-0.04716	-0.01692	0.145649	0.650896	0.257324	0.089255	0.168437	0.080092	0.082785	0.143443	0.050857
rsad2	0.859712	0.25798	-0.63007	-0.02834	1	0.197703	0.189495	0.105593	-0.26576	0.164119	0.588054	0.920071	0.811844	0.237092	0.767797	0.393452	0.757449	0.612679	0.080843	-0.35812	-0.49054
ifnc3	0.071066	0.288268	-0.04704	-0.09362	0.197703	1	0.48221	0.339722	-0.20524	0.576756	-0.07753	0.208898	-0.17065	-0.16446	0.236951	0.131229	-0.09347	-0.23981	0.251737	-0.06056	0.004216
tnf	0.027486	0.645835	-0.13686	-0.04708	0.189495	0.48221	1	0.333004	0.332428	0.803321	-0.29963	0.24414	-0.22409	0.288438	0.092724	0.015454	-0.17979	0.172778	0.356262	0.501475	0.267485
il1b	0.020968	0.234914	-0.16726	-0.32438	0.105593	0.339722	0.333004	1	-0.11897	0.452197	0.127373	0.012675	-0.11519	-0.29996	0.150625	-0.02563	-0.06594	-0.13211	0.169481	-0.08168	-0.1059
irf8	-0.14936	0.364865	0.346211	0.308945	-0.26576	-0.20524	0.332428	-0.11897	1	0.293406	-0.38055	-0.04369	-0.41419	0.433329	-0.1538	-0.11549	-0.41252	0.115991	0.081032	0.86591	0.265089
mhc class II	0.050158	0.544349	-0.06263	-0.17581	0.164119	0.576756	0.803321	0.452197	0.293406	1	-0.08022	0.242464	-0.29363	0.229594	0.028977	-0.00202	-0.25959	-0.02562	0.498839	0.438726	0.13
gzma	0.76408	-0.37522	-0.11692	-0.04716	0.588054	-0.07753	-0.29963	0.127373	-0.38055	-0.08022	1	0.597807	0.687027	0.07802	0.656242	-0.21491	0.750102	0.391957	0.304107	-0.47037	-0.78269
rigi	0.883531	0.31021	-0.4588	-0.01692	0.920071	0.208898	0.24414	0.012675	-0.04369	0.242464	0.597807	1	0.693177	0.359118	0.801386	0.259255	0.6781	0.732406	0.156971	-0.09188	-0.48071
isg15	0.770092	-0.20241	-0.5029	0.145649	0.811844	-0.17065	-0.22409	-0.11519	-0.41419	-0.29363	0.687027	0.693177	1	0.249369	0.747098	0.175383	0.922815	0.65258	0.068695	-0.59506	-0.6999
tlr3	0.32294	0.223448	0.07937	0.650896	0.237092	-0.16446	0.288438	-0.29996	0.433329	0.229594	0.07802	0.359118	0.249369	1	0.252317	0.151083	0.236304	0.558608	0.238076	0.417378	0.055552
cxcl10	0.80199	0.0416	-0.32851	0.257324	0.767797	0.236951	0.092724	0.150625	-0.1538	0.028977	0.656242	0.801386	0.747098	0.252317	1	0.04423	0.787673	0.590218	0.234312	-0.28869	-0.67214
saa	0.091341	0.521815	-0.4387	0.089255	0.393452	0.131229	0.015454	-0.02563	-0.11549	-0.00202	-0.21491	0.259255	0.175383	0.151083	0.04423	1	0.091257	0.082594	-0.38646	-0.12943	0.355487
ifit5	0.812858	-0.2759	-0.25564	0.168437	0.757449	-0.09347	-0.17979	-0.06594	-0.41252	-0.25959	0.750102	0.6781	0.922815	0.236304	0.787673	0.091257	1	0.627608	0.126457	-0.56109	-0.69449
ifi44	0.632442	0.223173	-0.25918	0.080092	0.612679	-0.23981	0.172778	-0.13211	0.115991	-0.02562	0.391957	0.732406	0.65258	0.558608	0.590218	0.082594	0.627608	1	0.125598	0.123654	-0.37849
irf1	0.019017	-0.1094	0.275263	0.082785	0.080843	0.251737	0.356262	0.169481	0.081032	0.498839	0.304107	0.156971	0.068695	0.238076	0.234312	-0.38646	0.126457	0.125598	1	0.071684	-0.46357
csf1r	-0.26926	0.558338	0.350935	0.143443	-0.35812	-0.06056	0.501475	-0.08168	0.86591	0.438726	-0.47037	-0.09188	-0.59506	0.417378	-0.28869	-0.12943	-0.56109	0.123654	0.071684	1	0.509698
**PRV3 dCt**	-0.58573	0.496761	0.100495	0.050857	-0.49054	0.004216	0.267485	-0.1059	0.265089	0.13	-0.78269	-0.48071	-0.6999	0.055552	-0.67214	0.355487	-0.69449	-0.37849	-0.46357	0.509698	1

Table S14. P-value of Pearson's correlation analysis of 18°C PRV-3 exposed shedders (based on all time points).

	mx	*cd4*	*cd8*	*ifng*	*rsad2*	*ifnc3*	*tnf*	*il1b*	*irf8*	*mhc class II*	*gzma*	*rigi*	*isg15*	*tlr3*	*cxcl10*	*saa*	*ifit5*	*ifi44*	*irf1*	*csf1r*	**PRV3 dCt**
mx	0	0.964422	0.181941	0.801844	1.96E-05	0.79368	0.919516	0.938563	0.580878	0.853644	0.000569	5.7E-06	0.000483	0.222466	0.000186	0.736547	0.000129	0.008565	0.94427	0.313238	0.017123
cd4	0.964422	0	0.157523	0.831264	0.334707	0.278928	0.006882	0.381148	0.16467	0.029254	0.152118	0.242277	0.452183	0.405475	0.878424	0.038154	0.300988	0.406068	0.68671	0.024588	0.050293
cd8	0.181941	0.157523	0	0.275266	0.008895	0.862664	0.613269	0.535811	0.188988	0.817765	0.666304	0.073854	0.047083	0.770142	0.214133	0.089154	0.339258	0.332382	0.302145	0.18262	0.711149
ifng	0.801844	0.831264	0.275266	0	0.917014	0.730212	0.862542	0.220295	0.244303	0.51486	0.862305	0.9504	0.590423	0.006319	0.335981	0.74237	0.532906	0.768106	0.760516	0.596123	0.851625
rsad2	1.96E-05	0.334707	0.008895	0.917014	0	0.462975	0.482116	0.697127	0.319806	0.543609	0.016581	4.5E-07	0.000134	0.37662	0.000515	0.131623	0.000678	0.011628	0.765988	0.173205	0.053707
ifnc3	0.79368	0.278928	0.862664	0.730212	0.462975	0	0.058545	0.197969	0.445734	0.019341	0.775338	0.437491	0.527446	0.542767	0.376911	0.628074	0.730625	0.371006	0.346943	0.823692	0.987636
tnf	0.919516	0.006882	0.613269	0.862542	0.482116	0.058545	0	0.207556	0.208391	0.000178	0.25956	0.362167	0.404097	0.278631	0.732691	0.9547	0.505223	0.522244	0.17561	0.047815	0.316558
il1b	0.938563	0.381148	0.535811	0.220295	0.697127	0.197969	0.207556	0	0.660774	0.07865	0.638295	0.962842	0.670981	0.259011	0.57765	0.924949	0.808303	0.625744	0.530333	0.763639	0.696287
irf8	0.580878	0.16467	0.188988	0.244303	0.319806	0.445734	0.208391	0.660774	0	0.270061	0.145913	0.872363	0.110719	0.093596	0.569559	0.670157	0.112308	0.668814	0.765455	1.46E-05	0.321079
mhc class II	0.853644	0.029254	0.817765	0.51486	0.543609	0.019341	0.000178	0.07865	0.270061	0	0.767741	0.365577	0.269673	0.392335	0.915163	0.99407	0.331596	0.924966	0.049189	0.089131	0.631323
gzma	0.000569	0.152118	0.666304	0.862305	0.016581	0.775338	0.25956	0.638295	0.145913	0.767741	0	0.014454	0.00328	0.773958	0.005765	0.4241	0.000818	0.133228	0.252152	0.065962	0.000338
rigi	5.7E-06	0.242277	0.073854	0.9504	4.5E-07	0.437491	0.362167	0.962842	0.872363	0.365577	0.014454	0	0.002908	0.171926	0.00019	0.332239	0.003888	0.001253	0.561531	0.735052	0.05945
isg15	0.000483	0.452183	0.047083	0.590423	0.000134	0.527446	0.404097	0.670981	0.110719	0.269673	0.00328	0.002908	0	0.351648	0.000881	0.515892	3.6E-07	0.00614	0.800432	0.01503	0.00254
tlr3	0.222466	0.405475	0.770142	0.006319	0.37662	0.542767	0.278631	0.259011	0.093596	0.392335	0.773958	0.171926	0.351648	0	0.345796	0.576482	0.378255	0.024503	0.374583	0.107724	0.838089
cxcl10	0.000186	0.878424	0.214133	0.335981	0.000515	0.376911	0.732691	0.57765	0.569559	0.915163	0.005765	0.00019	0.000881	0.345796	0	0.870796	0.000291	0.01609	0.382406	0.278196	0.004342
saa	0.736547	0.038154	0.089154	0.74237	0.131623	0.628074	0.9547	0.924949	0.670157	0.99407	0.4241	0.332239	0.515892	0.576482	0.870796	0	0.73678	0.761054	0.139248	0.632844	0.176618
ifit5	0.000129	0.300988	0.339258	0.532906	0.000678	0.730625	0.505223	0.808303	0.112308	0.331596	0.000818	0.003888	3.6E-07	0.378255	0.000291	0.73678	0	0.009247	0.640731	0.023741	0.002833
ifi44	0.008565	0.406068	0.332382	0.768106	0.011628	0.371006	0.522244	0.625744	0.668814	0.924966	0.133228	0.001253	0.00614	0.024503	0.01609	0.761054	0.009247	0	0.64302	0.648208	0.148299
irf1	0.94427	0.68671	0.302145	0.760516	0.765988	0.346943	0.17561	0.530333	0.765455	0.049189	0.252152	0.561531	0.800432	0.374583	0.382406	0.139248	0.640731	0.64302	0	0.791922	0.070518
csf1r	0.313238	0.024588	0.18262	0.596123	0.173205	0.823692	0.047815	0.763639	1.46E-05	0.089131	0.065962	0.735052	0.01503	0.107724	0.278196	0.632844	0.023741	0.648208	0.791922	0	0.043712
**PRV3 dCt**	0.017123	0.050293	0.711149	0.851625	0.053707	0.987636	0.316558	0.696287	0.321079	0.631323	0.000338	0.05945	0.00254	0.838089	0.004342	0.176618	0.002833	0.148299	0.070518	0.043712	0

1.1 Figures

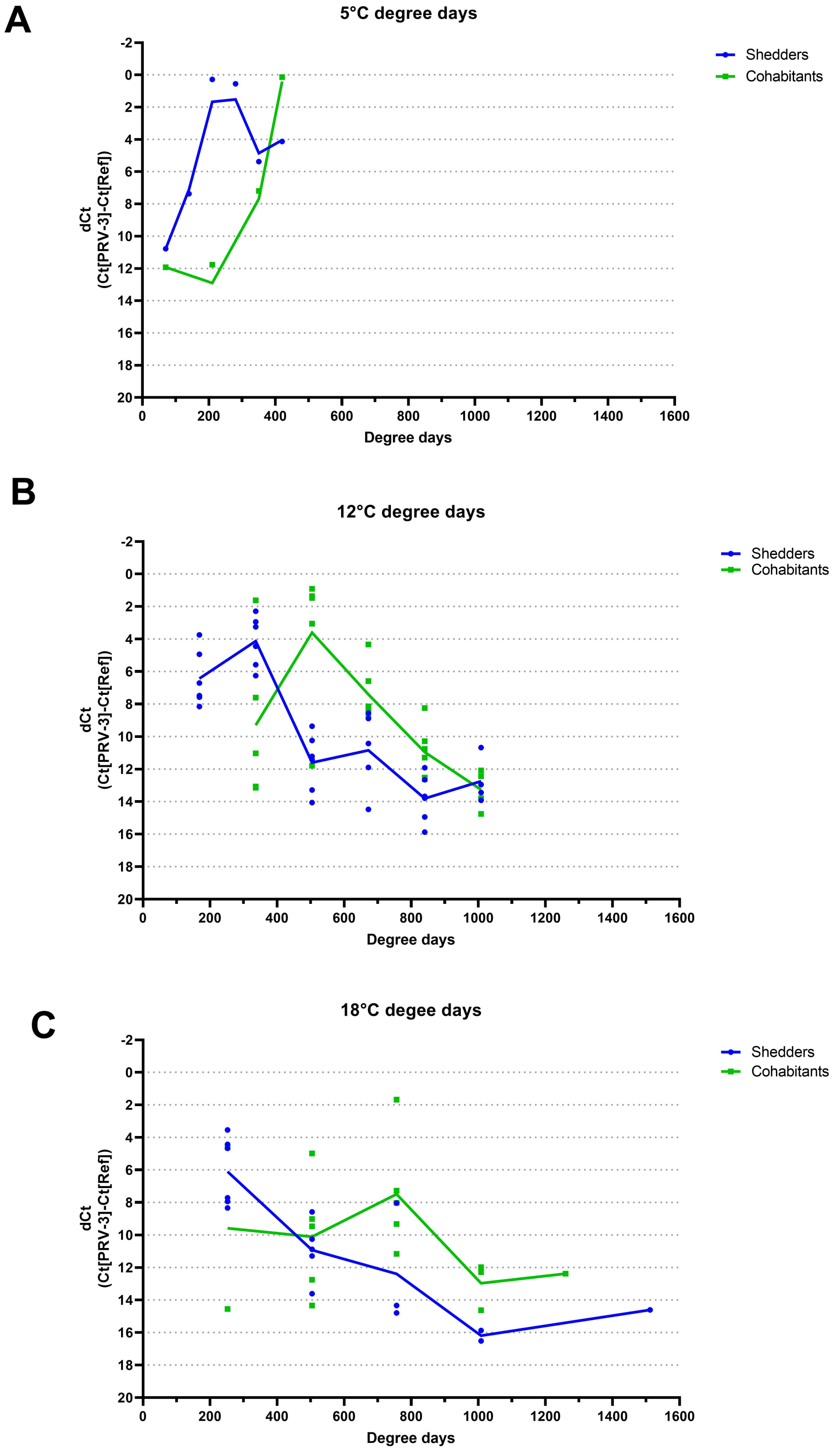

Figure S1. PRV-3 virus load across the trial shown as degree days. A) 5°C, B) 12°C, and C) 18°C, with green and blue representing cohabitants and shedders, respectively.

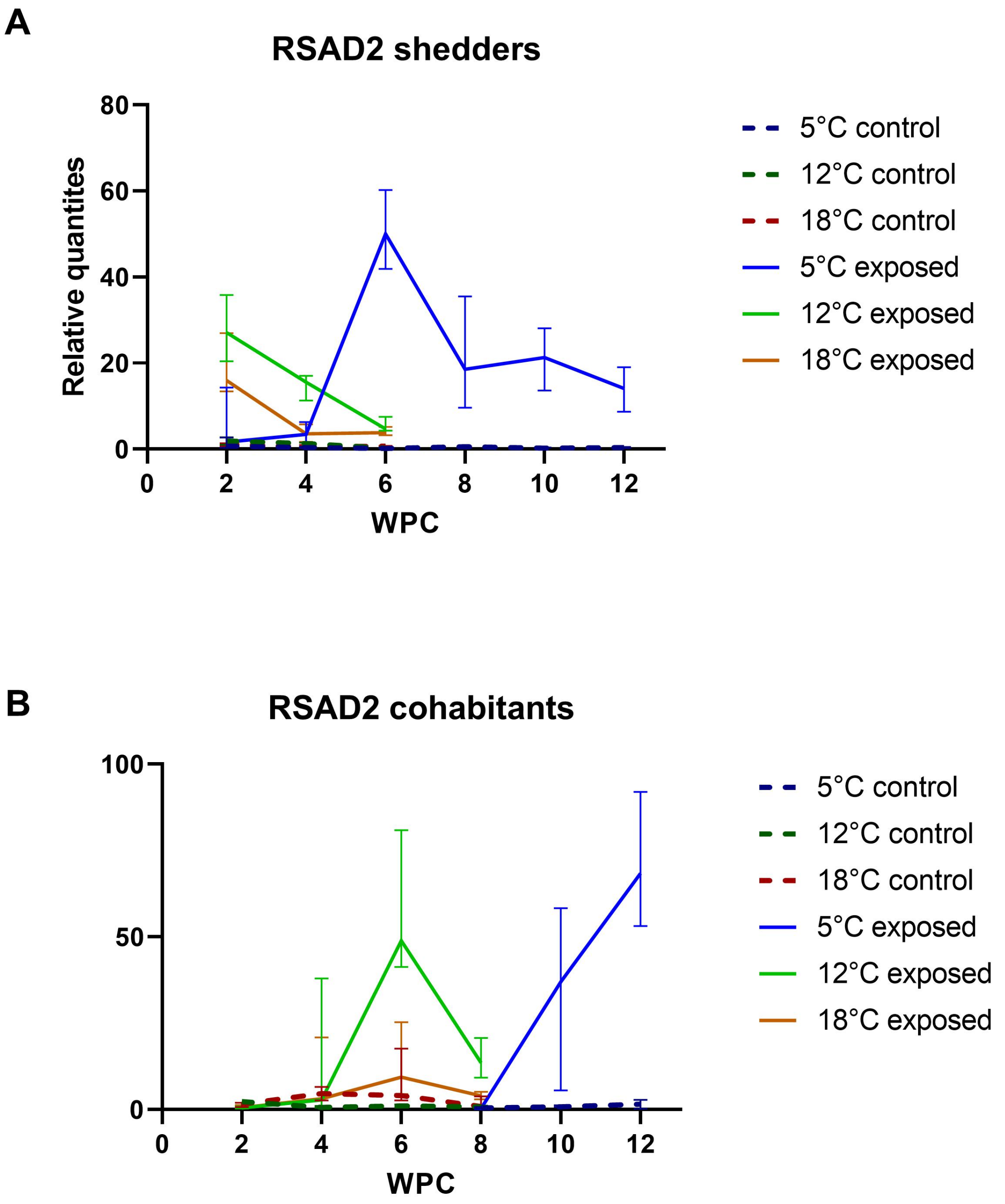

Figure S2. Relative quantities of *rsad2* (viperin) across the experiment in A) shedders and B) cohabitants. Blue: 5°C, green: 12°C, and orange: 18°C. Dotted line representing control fish, solid line representing PRV-3 exposed fish.

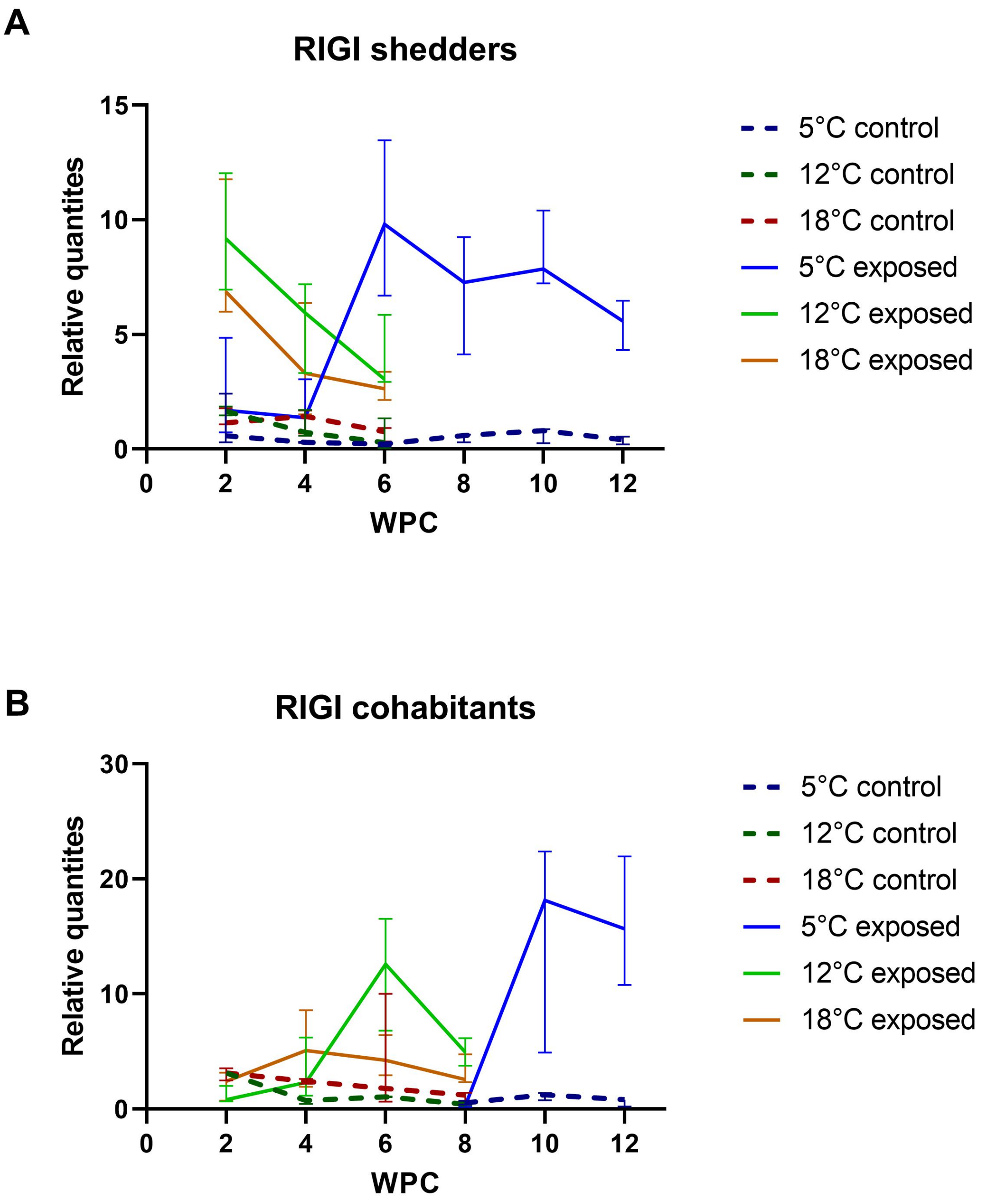

Figure S3. Relative quantities of *rigi* across the experiment in A) shedders and B) cohabitants. Blue: 5°C, green: 12°C, and orange: 18°C. Dotted line representing control fish, solid line representing PRV-3 exposed fish.

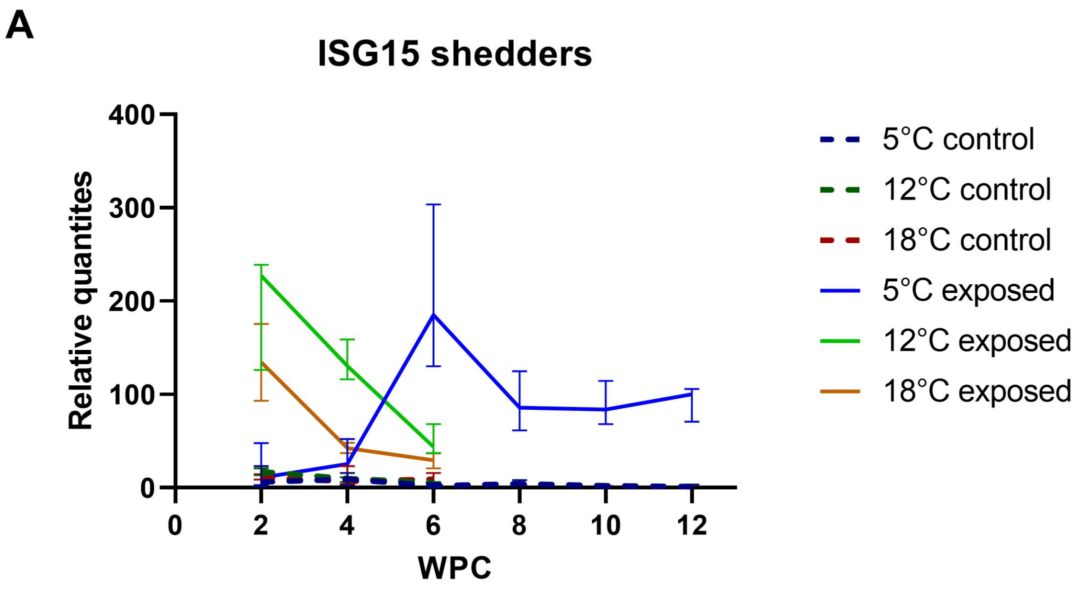

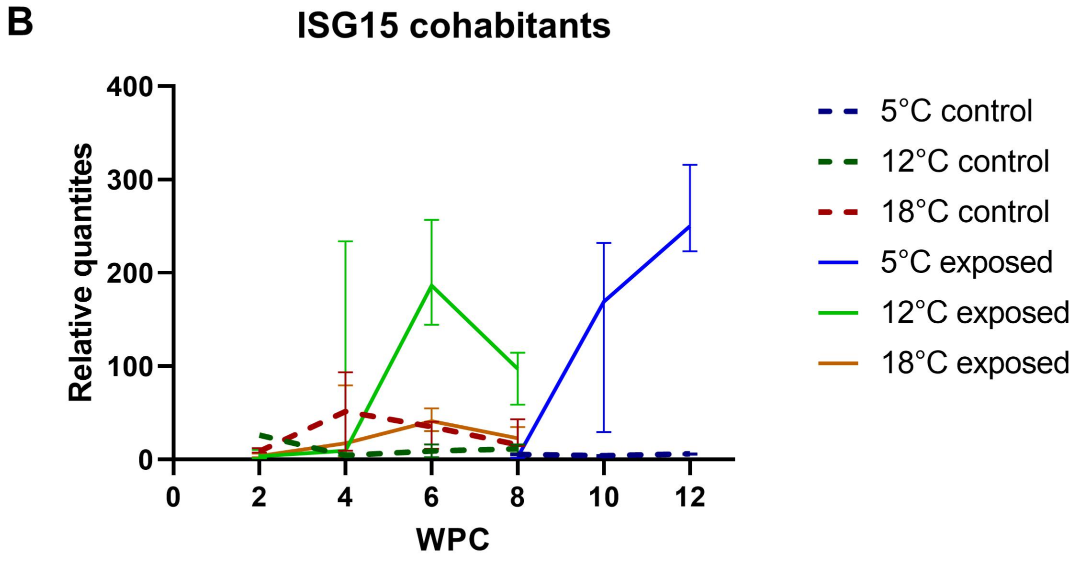

Figure S4. Relative quantities of *isg15* across the experiment in A) shedders and B) cohabitants. Blue: 5°C, green: 12°C, and orange: 18°C. Dotted line representing control fish, solid line representing PRV-3 exposed fish.

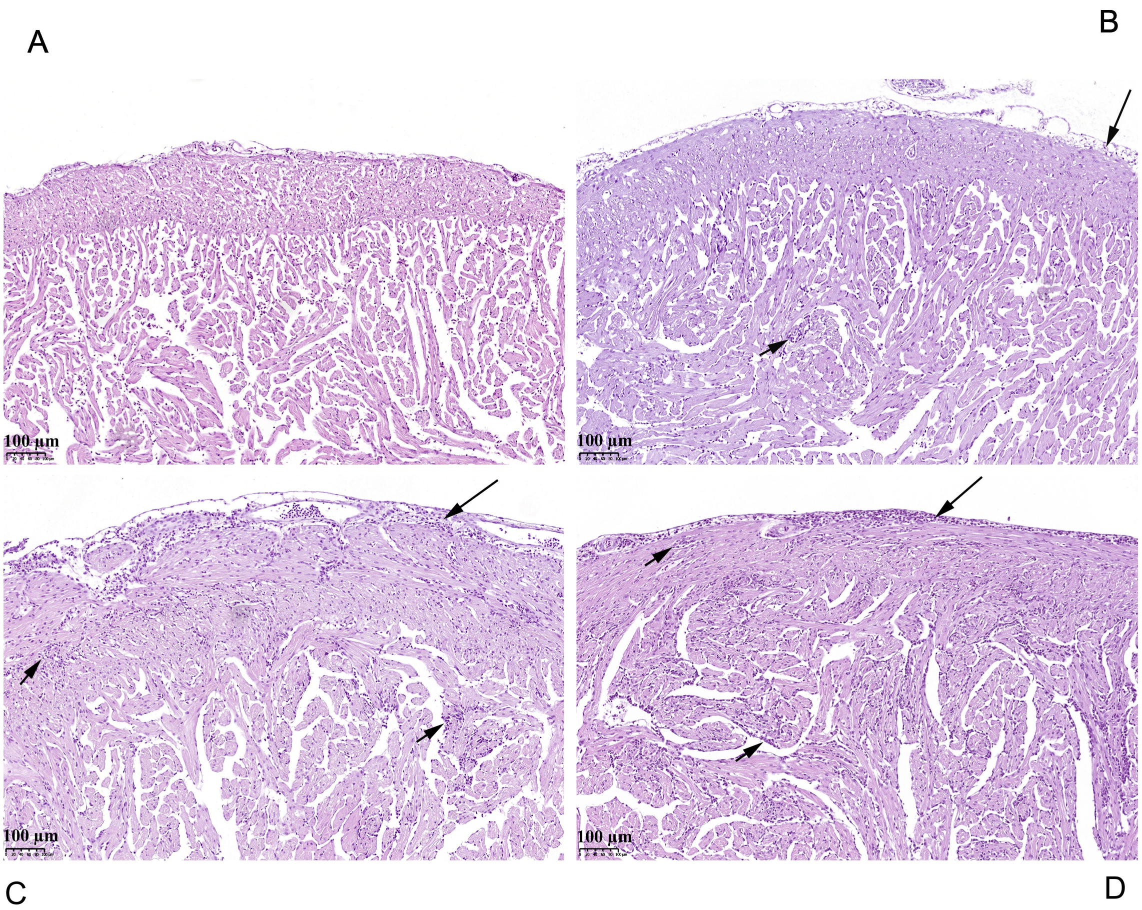

Figure S5. Histopathological scoring of the rainbow trout heart. A) no lesions, score 0; B) mild, score 1; C) moderate, score 1.5; D) severe, score 2. Epicarditis (long arrow) and inflammation in the stratum compactum (outer layer) and stratum spongiosum (internal layer) of the heart ventricle (short arrow) (H&E).
